# *Vibrio natriegens* genome‐scale modeling reveals insights into halophilic adaptations and resource allocation

**DOI:** 10.15252/msb.202110523

**Published:** 2023-02-27

**Authors:** Lucas Coppens, Tanya Tschirhart, Dagmar H Leary, Sophie M Colston, Jaimee R Compton, William Judson Hervey, Karl L Dana, Gary J Vora, Sergio Bordel, Rodrigo Ledesma‐Amaro

**Affiliations:** ^1^ Department of Bioengineering and Imperial College Centre for Synthetic Biology Imperial College London London UK; ^2^ US Naval Research Laboratory Center for Bio/Molecular Science and Engineering Washington DC USA; ^3^ NOVA Research Inc Alexandria VA USA; ^4^ Department of Chemical Engineering and Environmental Technology, School of Industrial Engineering University of Valladolid Valladolid Spain

**Keywords:** genome‐scale metabolic modeling, metabolic engineering, resource allocation, synthetic biology, *Vibrio natriegens*, Computational Biology, Metabolism, Microbiology, Virology & Host Pathogen Interaction

## Abstract

*Vibrio natriegens* is a Gram‐negative bacterium with an exceptional growth rate that has the potential to become a standard biotechnological host for laboratory and industrial bioproduction. Despite this burgeoning interest, the current lack of organism‐specific qualitative and quantitative computational tools has hampered the community's ability to rationally engineer this bacterium. In this study, we present the first genome‐scale metabolic model (GSMM) of *V. natriegens*. The GSMM (iLC858) was developed using an automated draft assembly and extensive manual curation and was validated by comparing predicted yields, central metabolic fluxes, viable carbon substrates, and essential genes with empirical data. Mass spectrometry‐based proteomics data confirmed the translation of at least 76% of the enzyme‐encoding genes predicted to be expressed by the model during aerobic growth in a minimal medium. iLC858 was subsequently used to carry out a metabolic comparison between the model organism *Escherichia coli* and *V. natriegens*, leading to an analysis of the model architecture of *V. natriegens*' respiratory and ATP‐generating system and the discovery of a role for a sodium‐dependent oxaloacetate decarboxylase pump. The proteomics data were further used to investigate additional halophilic adaptations of *V. natriegens*. Finally, iLC858 was utilized to create a Resource Balance Analysis model to study the allocation of carbon resources. Taken together, the models presented provide useful computational tools to guide metabolic engineering efforts in *V. natriegens*.

## Graphical abstract

iLC858 is the first constructed and validated genome‐scale metabolic model for the fast‐growing bacterium *Vibrio natriegens*. This new model enabled the study of *V. natriegens* metabolism, halophilic adaptions, and resource analysis.

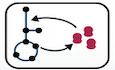

## Introduction

*Vibrio natriegens* is a Gram‐negative bacterium that in recent years has been gaining attention as an academically and industrially promising host for synthetic biology and biotechnology applications due to its exceptionally fast growth rate. The *V. natriegens* genome consists of two circular chromosomes, like the majority of members of the genus *Vibrio* (Hoff *et al*, 2020). Draft genomes were first made public in 2013, followed by the publication of two closed chromosomal contigs in 2016 (Maida *et al*, 2013; Wang *et al*, 2013; Lee *et al*, 2016). An automated annotation of the first completed genome by Lee *et al* (2016) resulted in the identification of 4,578 open reading frames and revealed the presence of 11 rRNA operons and 129 tRNA genes, markedly more than found in sister species *Vibrio cholerae* (8 rRNA operons and 98 tRNA genes) or *Escherichia coli* (7 rRNA operons and 99 tRNA genes). The annotation also suggested that *V. natriegens* has a versatile metabolism equipping it with the ability to grow on a vast range of carbon substrates, to reduce nitrate, and to fix atmospheric nitrogen under anaerobic, nitrogen‐limited conditions (Hoff *et al*, 2020). These properties enable it to thrive in coastal and estuarine waters and sediments—habitats characterized by fluctuations in salt concentration.

Although first isolated in 1958 and already highlighted for its remarkable generation time in 1962, interest in *V. natriegens* had waned in the intervening years but was rejuvenated in 2016 by the publication of two studies that provided genetic techniques for use in this organism and evaluated its potential as a laboratory workhorse (Payne, 1958; Eagon, 1962; Lee *et al*, 2016; Weinstock *et al*, 2016; Hoff *et al*, 2020). Specifically, Weinstock *et al* (2016) suggested *V. natriegens* as a time‐saving alternative to *E. coli* as a host for some of the most traditional laboratory applications such as molecular cloning and protein expression and secretion. Moreover, they demonstrated *V. natriegens'* ability to be cryopreserved up to 2 years and improved its tolerance to refrigeration, which are desirable properties for molecular cloning chassis. Similarly, Lee *et al* (2016) investigated optimal laboratory growth conditions, transformation protocols, and contributed plasmids to the genetic toolbox of *V. natriegens*. Since then, a number of groups have contributed to increasing the organism's genetic tractability (Dalia *et al*, 2017; Tschirhart *et al*, 2019; Wu *et al*, 2020).

In addition to interests in its potential as an alternative and potentially faster laboratory workhorse to *E. coli* for cloning and protein expression, *V. natriegens*' rapid growth has also sparked investigations into its potential as a host for industrial bioproduction. Hoffart *et al* (2017) highlighted the exceptionally high substrate uptake rate of *V. natriegens* as an attractive property for industrial bioconversions and fermentations. Additionally, they genetically engineered a strain to anaerobically produce alanine from glucose, producing titers that outperform streamlined *E. coli* and *Corynebacterium glutamicum* alanine production strains. Furthermore, a clever new genetic engineering method called MuGENT was designed to facilitate metabolic engineering for bioproduction requiring multiple genomic edits. This technique was subsequently leveraged to increase *V. natriegens*' natural ability to accumulate the bioplastic precursor poly‐β‐hydroxybutyrate (PHB) (Chien *et al*, 2007; Dalia *et al*, 2017). Finally, *V. natriegens* has been shown to be a viable production chassis for melanin production (Wang *et al*, 2020), production of natural products (Ellis *et al*, 2019), and a growth‐supporting auxin producer in cocultures with microalgae (Kim *et al*, 2019).

Given the heightened interest, rapid progress to date, and potential of *V. natriegens* as a biotechnological chassis, the field is now in need of computational tools to support its expansion into industrially relevant applications. One computational tool that facilitates the rational manipulation of metabolism is a genome‐scale metabolic modeling. A genome‐scale metabolic model (GSSM) is a mathematical reconstruction containing all the known metabolites, enzymatic metabolic reactions, and the corresponding genes for a certain organism (Orth *et al*, 2010). These genome‐scale representations provide a computational framework to mechanistically and quantitatively relate an organism's substrate uptake rates (in mmols) to biomass formation (in grams of dry cell weight), thereby enabling the *in silico* prediction of genotype–phenotype relationships (Orth *et al*, 2011; Chan *et al*, 2017). GSMMs are useful for a wide array of academic and industrial objectives. Examples of GSMM‐driven academic research include the prediction and optimization of growth on various substrates (Ibarra *et al*, 2002), analysis and interpretation of ‘‐omics’ data (Aurich *et al*, 2016), and prediction of metabolic demand adaptation in changing environments (Zuñiga *et al*, 2018). Within an application‐oriented context, GSMMs can be utilized for the design of metabolic pathways to produce non‐native compounds (Campodonico *et al*, 2014) and for the assessment of the effect of substrate‐related redox balances on biomass production (Contador *et al*, 2015). A minimal flux analysis model of the central metabolism, containing 42 reactions, had already been presented by Long *et al* (2017). In contrast, the scope of a GSMM goes far beyond the central metabolism, as they aim to represent as many of the enzymes encoded on an organism's genome as possible. This makes GSMMs useful tools for analysis of noncentral pathways, substrate viability prediction, and gene essentiality analysis, which cannot be done with metabolic models limited to central metabolism.

In this study, we present the first GSMM for *V. natriegens*. The model was constructed using an automated draft assembly followed by extensive manual curation and has been validated by comparing predicted phenotypes such as growth rates, fluxes through central metabolism, viable carbon substrates, and predicted essential genes to experimental data. In addition, the model allowed us to shed light on the mechanisms behind fast aerobic growth in saline environments and identified sodium‐dependent oxaloacetate decarboxylase as a potential enabler. The generation and analysis of proteomics data allowed to further elucidate *V. natriegens'* adaptations to increased salinity as well as the construction of a Resource Balance Analysis model.

## Results and Discussion

### Construction of the GSMM

**Figure 1:**
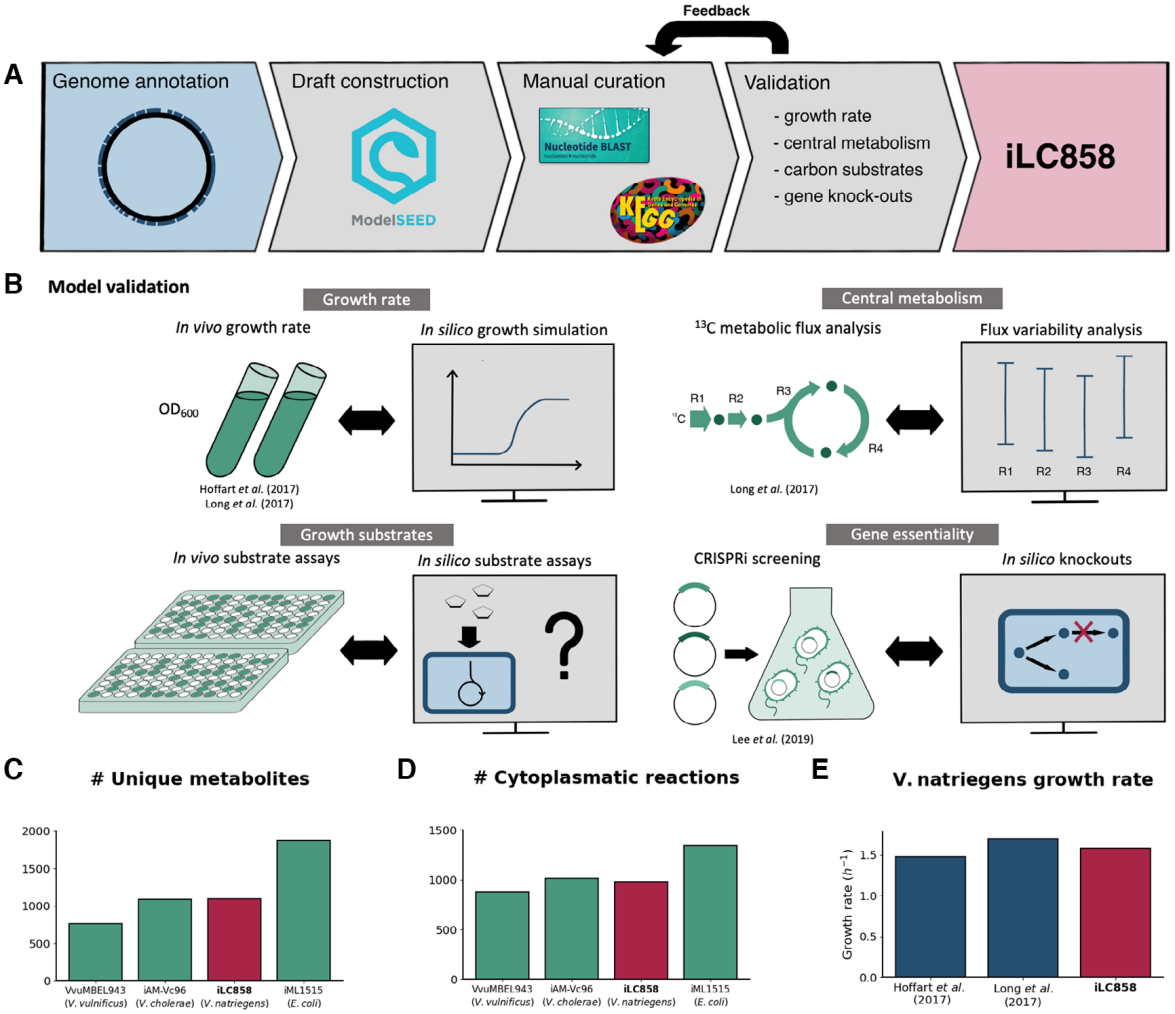
Construction and validation of iLC858 Flowchart of the construction process of iLC858.The different approaches taken for the validation of iLC858.Comparison of the number of unique metabolites in iLC858 with other *Vibrio* models and the model *E. coli* model iML1515.Comparison of the number of cytoplasmatic reactions in iLC858 with other *Vibrio* models and the model *E. coli* model iML1515.Comparison of the *in silico* growth rate of *V. natriegens* with reported *in vivo* growth rate on minimal glucose medium (Hoffart *et al*, 2017; Long *et al*, 2017).

Predicted coding sequences were extracted from the annotated genome of *V. natriegens* ATCC 14048 (NCBI accession CP009977.1 and CP009978.1) and subsequently used to generate a draft model using the SEED server (Overbeek *et al*, 2005). Missing reactions were manually added to the network based on knowledge retrieved from the genome annotation, NCBI BLAST (Altschul *et al*, 1990) and the KEGG (Kanehisa *et al*, 2012) database. The biomass reaction suggested by the SEED server was kept in the model. All reactions that had been included by the SEED server of which no equivalents were found in the *V. cholerae* model iAM‐Vc960 (Abdel‐Haleem *et al*, 2020) were manually checked and curated. Further curation of the model was performed during model validation (Fig 1A and B). A list of the performed manual curations can be found in Dataset EV1.

The vast majority (90.1%) of reactions in our model, not counting passive transmembrane transport reactions or metabolite sink reactions, are associated with at least one known coding sequence in the *V. natriegens* genome. A mapping was done of metabolite, reaction, and gene ids to identifiers across online biochemistry databases to ensure cross‐database compatibility. The quality of the GSMM was assessed using the MEMOTE software suite, which has been developed as a benchmarking and quality control tool for GSMMs that also assesses cross‐compatibility with various databases (Lieven *et al*, 2020). The MEMOTE software reported a score of 90%, which is comparable to the 91% yielded by MEMOTE for both the benchmark *Escherichia coli* model iJO1366 (Orth *et al*, 2011; Monk *et al*, 2017) and iML1515 (Monk *et al*, 2017). A breakdown of this score can be found in Dataset EV2.

Excluding exchange and transport reactions, the resulting GSMM iLC858 consists of 1,096 unique metabolites connected by 982 cytoplasmatic reactions. This makes iLC858 more extensive than the *Vibrio vulnificus* model VvuMBEL943 (Kim *et al*, 2011), which contains 761 unique metabolites and 878 cytoplasmatic reactions, and a similar size to *Vibrio cholerae* model iAM‐Vc960 (Abdel‐Haleem *et al*, 2020), which contains 1,087 unique metabolites and 1,016 cytoplasmatic reactions (Fig 1C and D). We also performed ID mapping of reactions from iLC858 to iAM‐Vc960 and have included this as Dataset EV3. Compared to the highly curated *E. coli* model iML1515, iLC858 is still limited in size: only 858 *V. natriegens* genes were accounted for in our model, compared to the 1,515 *E. coli* genes accounted for in iML1515.

### Biomass yield on minimal medium

We first simulated aerobic growth on minimal medium by providing glucose as the sole carbon source in the *in silico* medium. When solving a GSMM using Flux Balance Analysis (FBA), the model will maximally utilize any provided sources of carbon to generate biomass. Hence, constraints on substrate uptake rate and secretion of metabolites must be imposed to generate realistic simulations.

Even under aerobic conditions, *V. natriegens* secretes part of its substrate as acetate, indicating a mixed respiratory‐fermentative growth phenotype. This behavior is similar to the Crabtree effect observed in yeast under aerobic conditions, which is hypothesized to be a reflection of the fitness trade‐off between the efficiency gained by full respiration and the cost of the expression of the respiratory pathways (Molenaar *et al*, 2009). The glucose uptake rate of *V. natriegens* during aerobic growth on minimal medium was independently experimentally determined to be 3.9 g gDW^−1^ h^−1^ by Hoffart *et al* (2017) and Long *et al* (2017). We used this value to constrain the substrate uptake rate in simulations of growth on minimal glucose medium. After constraining *in silico* acetate secretion rate to 1.4 g gDW^−1^ h^−1^, a value found in Hoffart's data during exponential growth on minimal medium, a growth rate of 1.59 h^−1^ was obtained. This growth rate, which reflects the partial fermentative behavior of *V. natriegens*, is in good agreement to growth rates of 1.48 and 1.7 h^−1^ experimentally determined by Hoffart *et al* (2017) and Long *et al* (2017), respectively (Fig 1E). Using these stoichiometries for glucose and acetate exchange, our model predicts a biomass yield of 0.41 grams per gram of consumed glucose, nearly matching the reported biomass yield of 0.38 grams per gram of consumed glucose reported by Hoffart *et al* (2017) (0.38 g g^−1^).

### Prediction of fluxes through central metabolism

Pathways such as glycolysis, pentose phosphate pathway (PPP), and the tricarboxylic acid cycle (TCA) form the central motor driving metabolism in bacteria. Given the central role these pathways play in metabolism during growth, we investigated how well the predicted corresponding fluxes compared to experimentally determined central metabolic fluxes. We simulated aerobic growth on glucose minimal medium using iLC858, and compared the fluxes to data reported by Long *et al* (2017), who used ^13^C metabolic flux analysis to map fluxes through glycolysis, PPP, TCA, the Entner–Doudoroff (ED) pathway, and the glyoxylate shunt (GS). For this simulation, the same glucose uptake rate and acetate secretion rate as in the aerobic growth simulations were used. The predicted fluxes were represented as percentages of the glucose uptake rate, in order to enable comparison to Long's data. Comparing the fluxes predicted by the model's optimal solution and experimental fluxes yielded a high Pearson's correlation coefficient of 99.2%, suggesting high consistency between our model and *V. natriegens'* central metabolism.

**Figure 2:**
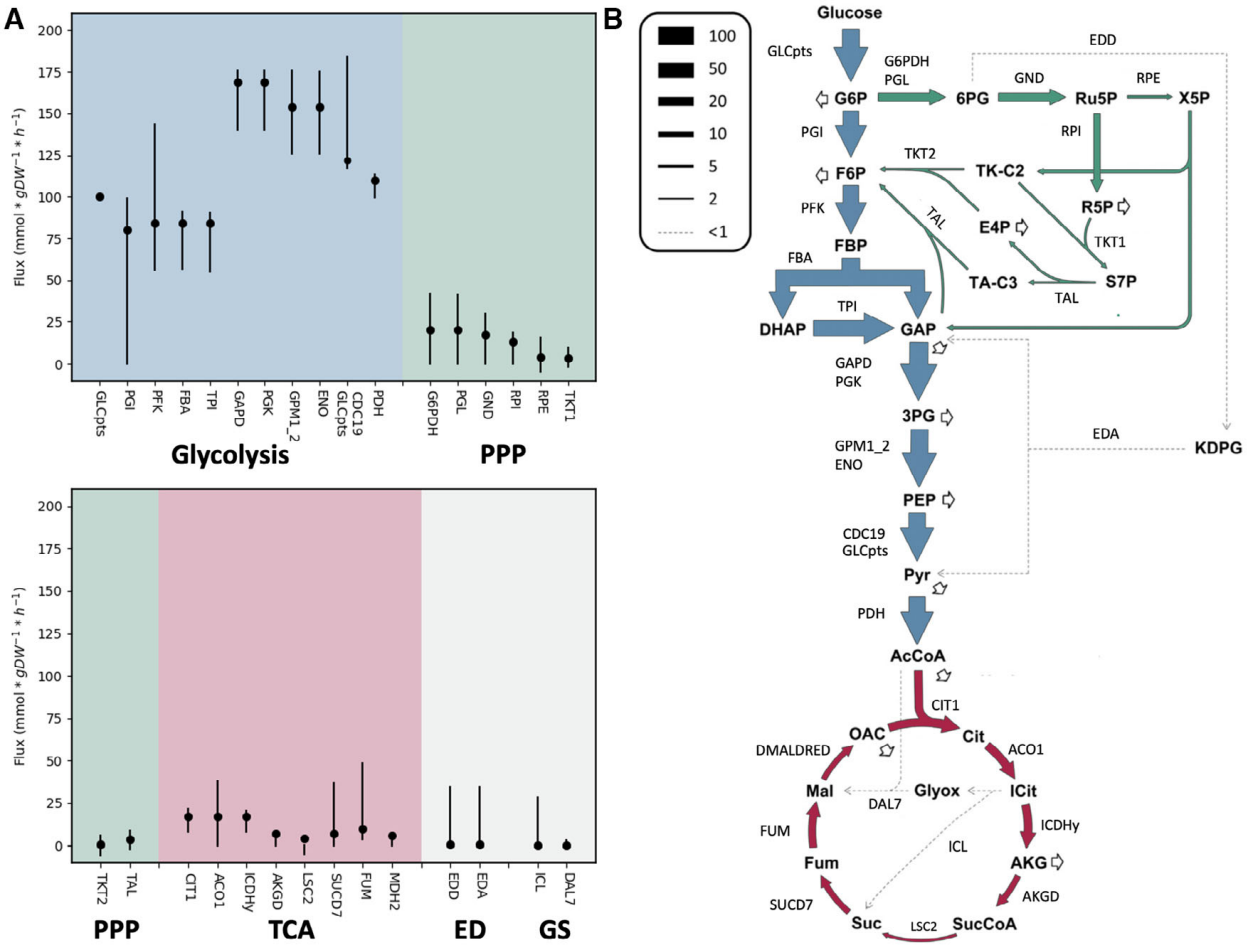
FVA results and experimentally determined fluxes through *Vibrio natriegens'* central metabolism Dots indicate experimentally determined fluxes by Long *et al* (2017), vertical lines indicate the FVA‐determined intervals for each reaction at which *in silico* growth remains above 98% of the optimal solution. Reactions are grouped and colored per subsystem: glycolysis, pentose phosphate pathway (PPP), TCA (tricarboxylic acid cycle), Entner–Doudoroff pathway (ED), and glyoxylate shunt (GS).Schematic overview of fluxes through *V. natriegens'* central metabolism with corresponding reaction codes. Adapted from Long *et al* (2017) (colorization of the arrows).

Additionally, we simulated the fluxes through the central metabolism using flux variability analysis (FVA), producing boundaries on these fluxes allowing solutions yielding at least 98% of the optimal solution in terms of produced biomass. The results of this comparison, together with a schematic overview of experimentally determined fluxes through central metabolism, are shown in Fig 2A and B. This visualization again demonstrates high correlation between the model's preferred fluxes near the optimal solution and the metabolic fluxes determined by Long *et al* (2017).

Long *et al* (2017) posited that while many marine bacteria prefer ED over glycolysis, their data confirmed that, like for most members of the genus *Vibrio*, this is not the case in *V. natriegens*, which has a near‐zero flux through ED. Interestingly however, the model is flexible when it comes to carbon passing through the ED pathway as an alternative to glycolysis, indicating that the machinery for efficient glucose utilization through the ED pathway is encoded within the *V. natriegens* genome. This is consistent with early findings done by Eagon & Wang (1962) who detected the enzymes of the ED pathway in *V. natriegens* when grown on gluconate. Similarly, it has been shown that the ED pathway is activated in *E. coli* when it is grown on gluconate (Eisenberg & Dobrogosz, 1967).

### Growth on different substrates

Carbon substrate uptake and utilization represent critical bottlenecks in industrial bioconversion processes and are, therefore, interesting properties to study in *V. natriegens* using a GSMM.

**Figure 3:**
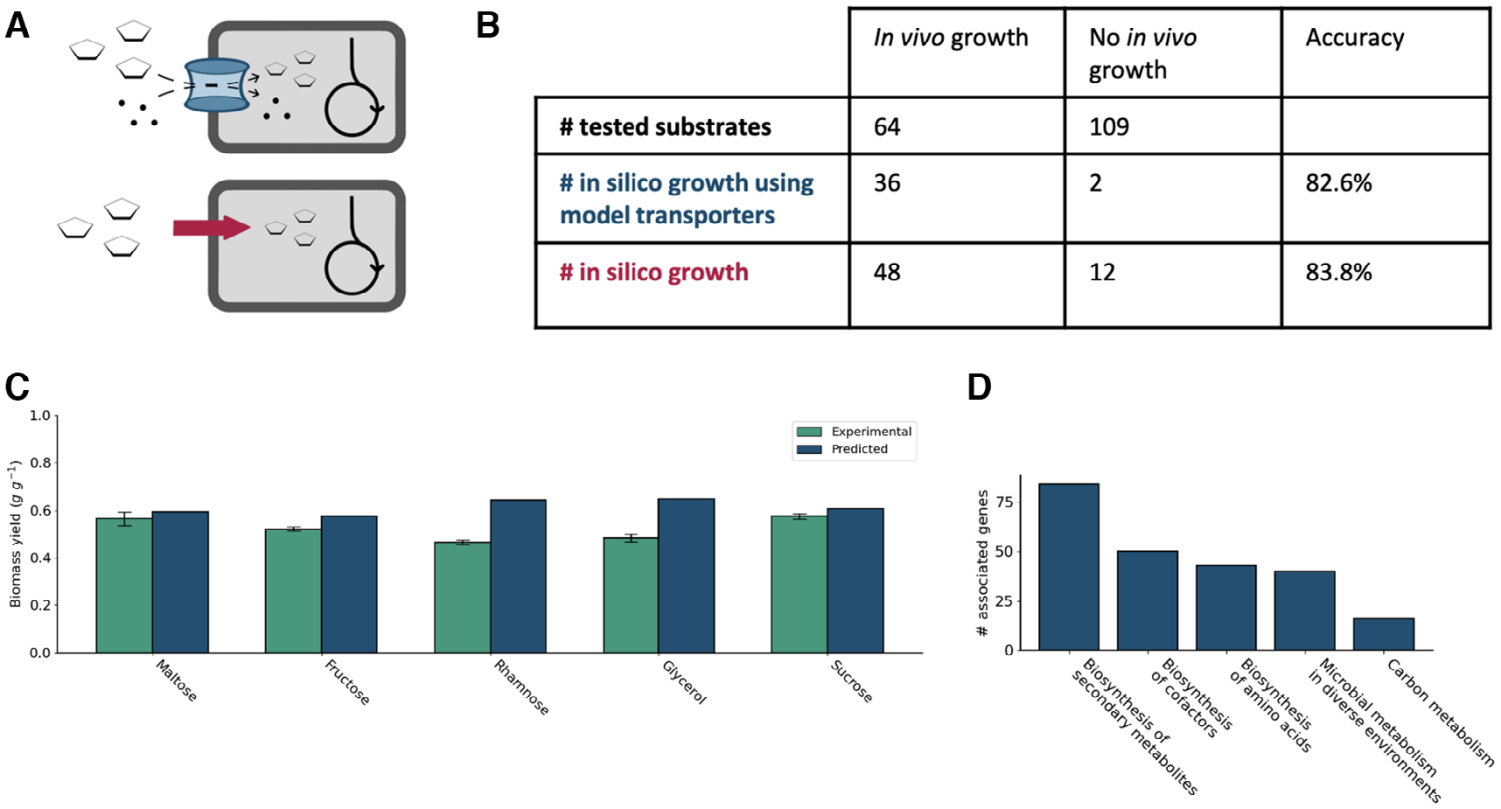
Carbon source viability and gene essentiality *In silico* carbon substrate tests can be performed either by supplying substrate to the extracellular space, which forces the model to use transporter reactions, or by directly supplying the cytoplasm with the substrate.Distribution of carbon sources showing *in vivo* or no *in vivo* growth across the two methods of *in silico* growth viability evaluation mentioned in A.Comparison of experimental vs. predicted yields on 5 carbon substrates. Error bars represent the standard deviation across three biological replicates.Ontologies associated with predicted essential genes.

We experimentally assessed *V. natriegens'* ability to use 173 distinct carbon sources included in iLC858 using BioLog Phenotype MicroArrays, a high‐throughput assay which measures respiration as a proxy for growth. 64 of 173 were found to be viable carbon substrates for *V. natriegens*. Subsequently, *in silico* growth prediction was determined for each substrate by creating an extracellular source, which forces the model to use its transporters, and alternatively, by providing a direct cytoplasmic supply (Fig 3A and B). An overview containing all experimentally tested substrates, including those that are not in the model, can be found in Dataset EV4. The model predicted growth for 48 of 64 (75%) carbon sources on which *V. natriegens* can grow and predicted nonviability for 97 of 109 (89%) carbon sources on *which V. natriegens* cannot grow, leading to an overall predictive accuracy of 83.8%.

While these numbers show good correlation between the *in silico* predictions and experimental data, we suggest a few explanations for the discrepancies between prediction and experimentation in addition to what is discussed above. Firstly, it is important to notice that the experimental data quantify respiration and not growth, and some of these substrates, while potentially used for cellular respiration, may not be enough to allow biomass synthesis. Secondly, homology‐based functional annotation of substrate‐specific transport proteins is a notoriously complicated task (Gelfand & Rodionov, 2008; Pelicaen *et al*, 2019). Some transporters may have been inaccurately annotated in the *V. natriegens* genome, leading to both a lack of annotated transporters for carbon sources *V. natriegens* can grow on and the presence of annotated transporters in the model for carbon sources *V. natriegens* is not capable of growing on. Similarly, many enzymes act on a variety of substrates through promiscuous activity, which often limits the comprehensiveness of GSMMs (Amin *et al*, 2019). These promiscuous enzymes could be a major source of metabolic reactions that are missing in our model. Furthermore, it is also possible that while the machinery required to metabolize certain substrates has been annotated and included in the model, the corresponding genes are not sufficiently expressed to enable growth, as was shown to be the case for xylose‐based growth in *S. cerevisiae* (Scalcinati *et al*, 2012). Finally, it could be that high concentrations of certain substrates are toxic for *V. natriegens* even though growth could be possible at lower concentrations—as is the case for *A. gossypii* growth on D‐ribose (Ledesma‐Amaro *et al*, 2014). This could have led to impaired *V. natriegens* growth in our experimental assays which is an effect that GSMMs cannot account for.

Biomass yield per unit of carbon substrate represents a critical parameter in industrial bioconversion processes and is, therefore, a topic of interest in *V. natriegens*. We curated our GSMM with particular attention to commonly used substrates maltose, fructose, rhamnose, glycerol, and sucrose and experimentally measured the biomass yields for each of these substrates. A comparison of experimental and predicted biomass yields is provided in Fig 3C. The predicted yields match the experimental data well for sugars maltose, fructose, and sucrose but overestimate the yields for rhamnose and glycerol. This might be due to the secretion of metabolic by‐products, similar to the secretion of acetate when *V. natriegens* is grown on glucose (Hoffart *et al*, 2017; Long *et al*, 2017). These results can potentially serve as a basis for investigations aimed at improving the utilization of these carbon sources in *V. natriegens*. Ibarra *et al* (2002) provided a salient example of this approach when they unlocked the GSMM‐predicted potential of *E. coli* K‐12 to efficiently utilize glycerol, increasing its growth rate on glycerol from suboptimal to the growth rate predicted by their model using adaptive laboratory evolution.

Sucrose showed the highest experimental yield of the tested carbon sources (0.58 g g^−1^ experimental, 0.61 g g^−1^ predicted), being significantly higher than the biomass yield reported for glucose (0.38 g g^−1^, Hoffart *et al*, 2017). Moreover, sucrose was shown to have the highest *V. natriegens* growth rate on minimal medium from all the substrates tested by Hoffart *et al* (2017). In *E. coli*, sucrose as a carbon substrate for fermentations has also shown promise due to lower by‐production of lactic acid than glucose, resulting in higher biomass yields than glucose (0.58 g g^−1^, Arifin *et al*, 2014). These findings corroborate sucrose as an attractive carbon substrate for *V. natriegens*.

### Gene essentiality

GSMMs also provide a suitable platform to study gene essentiality*. In silico* knock out growth experiments were conducted with the model to evaluate the essentiality of every gene for growth on glucose. Knocking out 182 of 858 genes *in silico* resulted in an unsolvable FBA or growth rate reduced below 90% of the wild type growth rate, which we classified as “essential.” An overview of the ontologies of genes for which essentiality was predicted is provided in Fig 3D, and a list with the predicted essential genes is provided as Dataset EV5. Among these essential genes were five genes from the central metabolism, including genes encoding for phosphoglycerate kinase, glyceraldehyde‐3‐phosphate dehydrogenase, a gene encoding ribulose‐5‐phosphate 3‐epimerase, and two genes from the TCA cycle: citrate hydroxymutase and isocitrate:NADP+ oxidoreductase. Besides phosphoglycerate kinase and glyceraldehyde‐3‐phosphate dehydrogenase, no genes involved in glycolysis were classified as essential, likely because the model allows to employ the Entner–Doudoroff pathway as an alternative for glucose catabolism when glycolysis is unavailable.

Unfortunately, genome‐wide KO experiments have not been carried out in this organism, which limit our capacity to validate the model predictions. However, we compared our list of predicted essential genes with a high‐throughput CRISPRi screen performed by Lee *et al* (2019). A 171 out of the 858 *V. natriegens* genes incorporated in the model were suggested by Lee to be core genes. Of these, 83 were also found by the *in silico* analysis as “essential genes.” A confusion matrix of this comparison is provided as Appendix Table S1. However, it is worth mentioning that Lee's CRISPRi screens rely on putative reduced expression rather than true knockouts and a lack of exact reproducibility of the CRISPRi high‐throughput screen was reported by Lee *et al* (2016, 2019). Further discrepancies between Lee's dataset and the model's predictions on gene essentiality could be related to missing or inaccurate gene‐reaction associations in our model. Taken together, we anticipate that our model's predictions can be used to complement the Lee *et al* (2016, 2019) dataset in determining gene essentiality. Moreover, *in silico* gene essentiality can quickly predict genome‐wide gene essentiality in a variety of substrates and growth conditions. In future work, manual examination of the sets of nonoverlapping essential genes could provide insight into enzymes roles or specificities in *V. natriegens*.

### Expression of metabolic genes

We carried out a proteomics analysis on samples taken during exponential *V. natriegens* growth in minimal medium to further validate our model. We collected samples at two timepoints during *V. natriegens* exponential growth on minimal glucose medium at salinities of 0, 300, 540, and 650 mM NaCl. For comparison, the most commonly used *V. natriegens* cultivation medium LB3 contains 530 mM NaCl. The results are summarized in Dataset EV6. The export from Scaffold with the settings mentioned in the methods section was used. Normalized spectrum counts were used as a quantitative value for comparison among samples. We then studied if the enzymes predicted by the model were found experimentally.

Flux sampling was used to determine reactions in the GSMM which are predicted to be able to produce fluxes of at least 10^−6^ mmol g^−1^ h^−1^, a threshold taken from Altea‐Manzano *et al* (2020). Across 100 FBA samples of *V. natriegens* aerobic growth, 390 such reactions were identified. Of these, 300 reactions (76.9%) had associated genes of which the corresponding coded enzymes were entirely found in at least one of the proteomics samples. Moreover, 331 reactions (84.9%) had associated genes of which all enzymatic subunits except one were found in at least one of the proteomics samples.

### A metabolic comparison with model organism *E. coli*

The outstanding growth rate of V. natriegens inspired us to compare its metabolic network with the model organism *E. coli*. Mapping reactions from one GSMM to another allows to identify reactions that are shared between or unique to one of the metabolic networks. Hence, we performed a reaction mapping of all cytoplasmatic reactions in ILC858 to the *E. coli* GSMM iML1515 and found 772 shared reactions out of 1,187 *V. natriegens* cytoplasmatic reactions (65%, Dataset EV7).

**Figure 4:**
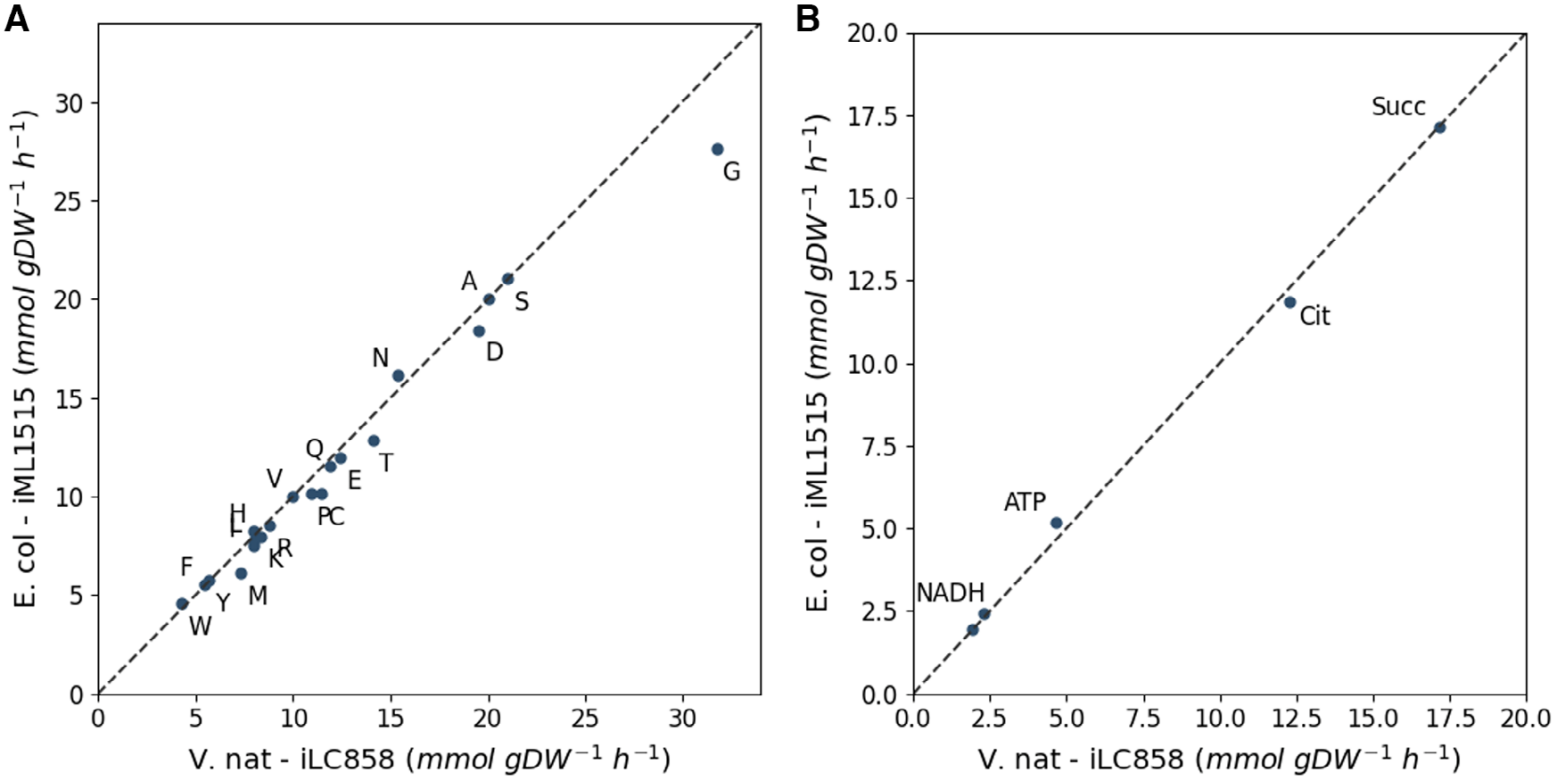
Comparison of maximal theoretical GSMM yields on 10 mmol glucose gDW‐1.h‐1 between iLC858 and iML1515 Comparison of maximal theoretical yields for all 20 amino acids between iLC858 and iML1515.Comparison of maximal theoretical yields for selected energy carriers and TCA intermediates between iLC858 and iML1515.

Subsequently, we compared our model iLC858 with the model iML1515 in their theoretical capacities to produce biomolecules. Figure 4A and B show comparisons of maximal theoretical yields for all 20 amino acids as well as some central molecules in energy metabolism. The comparison suggests that *V. natriegens*'s metabolic network has a higher theoretical capacity to produce most amino acids than *E. coli*. This difference is most notable for glycine. Hence, we looked for reactions that *V. natriegens*' metabolic network can leverage to produce glycine, that were not found in the reaction mapping to iML1515. We identified an alanine dehydrogenase and L‐Alanine:glyoxylate aminotransferase in iLC858, two enzymes which are not encoded in the *E. coli* genome. The L‐Alanine:glyoxylate aminotransferase was not identified in any of our proteomics samples and the alanine dehydrogenase was only found in low amounts at higher salinities. Transcripts encoding both enzymes, however, were found with high ribosomal density in a ribosomal profiling dataset generated by Lalanne *et al* (2018), where *V. natriegens* was grown in complete MOPS medium. This suggests that these two enzymes may provide a metabolic advantage to *V. natriegens* only under specific conditions.

We subsequently looked for *V. natriegens* reactions participating in biomass production that did not map to iML1515 but indicated expression in our proteomics dataset. An overview sorted by flux can be found as Dataset EV8. The previously identified alanine dehydrogenase and L‐Alanine:glyoxylate aminotransferase were among the reactions with the highest fluxes that were not found in *E. coli*. Na^+^‐translocating NADH:ubiquinone oxidoreductase was the reaction with the highest flux for biomass production in *V. natriegens* that was not found in *E. coli*. This enzyme is a pump commonly found in halophilic bacteria like *V. natriegens*. Additionally, a citrate hydroxymutase and Na^+^‐translocating oxaloacetate decarboxylase were found to have high fluxes.

Lastly, we compared the biomass reactions of iLC858 with *E. coli* models iJO1366 (Orth *et al*, 2011) and iML1515 (Monk *et al*, 2017). For the comparison, we selected all compounds from the *E. coli* biomass reactions in those models that could also be produced by our *V. natriegens* model. For each comparison, we respectively created artificial biomass reactions with only compounds that could be created by both iLC858 and the *E. coli* model in question. With these artificial biomass reactions and a glucose uptake rate of 3.9 g g^−1^ h^−1^, the iLC858 and iJO1366 predicted similar growth rates of 2.28 and 2.36 h^−1^, respectively. iLC858 and iML1515 predicted similar growth rates of 1.95 and 2.02 h^−1^. These simulations suggest that *V. natriegens* holds no obvious intrinsic metabolic advantage when compared to *E. coli*.

### Respiratory chain and halophilic adaptations

Given the fast growth rate and the halophilic lifestyle of *V. natriegens*, we curated the model with particular attention to the respiratory chain and corresponding ATP‐generating systems. The electron transport chain in *Vibrio* species is coupled to the pumping out of sodium through its primary respiratory pump, a Na^+^‐translocating NADH:quinone oxidoreductase (Na^+^‐NQR), in addition to pumping out protons using terminal oxidases. The resulting proton motive force (PMF) and sodium motive force (SMF) are interchangeable through sodium‐proton antiporters and drive processes such as the rotation of a flagellum and ATP synthesis through oxidative phosphorylation. The central role of Na^+^‐NQR and the mechanisms related to proton and sodium gradients in *V. cholerae* have been previously described in detail (Häse *et al*, 2001; Steuber *et al*, 2014, 2015).

Like *V. cholerae*, the genome of *V. natriegens* encodes for Na^+^‐translocating oxaloacetate decarboxylase (Na^+^‐OAD) (Dahinden *et al*, 2005). This pump has been shown to be reversible *in vitro*, allowing it to act as a pyruvate carboxylation driven by the SMF (Dimroth & Hilpert, 1984). It has been studied extensively in the context of fermentations with citrate as carbon source in the Gram‐negative bacteria *Klebsiella pneumoniae* and *Salmonella typhimurium* (Wifling & Dimroth, 1989; Xu *et al*, 2020). In contrast to *V. cholerae*, whose genome encodes for two Na^+^‐OAD gene sets of which one is located in the citrate fermentation operon (Dahinden *et al*, 2005), we only found one Na^+^‐OAD on the *V. natriegens* genome. *V. natriegens'* Na^+^‐OAD genes share highest homology with the *V. cholerae* Na^+^‐OAD that is not part of the citrate fermentation operon. In addition, it seems that the citrate fermentation operon, including Na^+^‐OAD, is entirely absent in *V. natriegens* (Table 1). These findings suggest a function unknown so far for this enzyme, that is not related to citrate fermentation.

**Table 1:** Homology of the two *Vibrio cholerae* NA^+^‐OAD gene clusters and citrate‐sodium symporter in *Vibrio natriegens*.

*V. cholerae* El Tor strain C6709 coordinates (Chr1)	Product	Function in *V. cholerae*	*V. natriegens* tblastn strongest hit (Chr1)	
2282892–2284238 (+)	Citrate‐sodium symporter	(Citrate) fermentation	No hit	0%
2284231–2284605 (+)	Na^+^‐OAD gamma chain	(Citrate) fermentation	No hit	0%
2284624–2286423 (+)	Na^+^‐OAD alpha chain	(Citrate) fermentation	236020–237798 (+) (PN96_01210)	65%
2286439–2287740 (+)	Na^+^‐OAD beta chain	(Citrate) fermentation	237811–238881 (+) (PN96_01215)	71%
2553183–2553443 (−)	Na^+^‐OAD gamma chain	Unknown	235724–235978 (+) (PN96_01205)	83%
2551361–2553148 (−)	Na^+^‐OAD alpha chain	Unknown	236014–237798 (+) (PN96_01210)	89%
2550221–2551351 (−)	Na^+^‐OAD beta chain	Unknown	237811–238881 (+) (PN96_01215)	97%

The citrate‐sodium symporter has no corresponding tblastn hit and the OAD that is not associated with the citrate fermentation operon is the closest homolog to the *V. natriegens* OAD. These results suggest a complete deletion of the citrate fermentation operon in *V. natriegens*.

**Figure 5:**
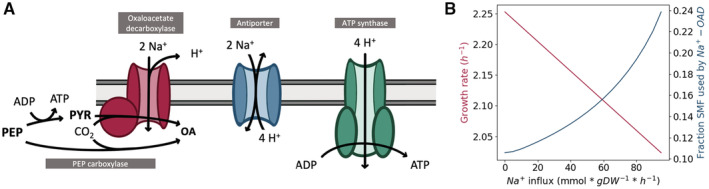
Role of the Na^+^‐OAD in *Vibrio natriegens* Schematic overview of part of the *V. natriegens* membrane system. Two extracellular Na^+^ ions can be used either to drive carboxylation of pyruvate to oxaloacetate or to generate one ATP through functions of the antiporter and ATP synthase. OA, oxaloacetate; PEP, phosphoenolpyruvate; PYR, pyruvate.Predicted growth rate and fraction of the SMF consumed by the Na^+^‐OAD as a function of increasing sodium ion influx.

The suggested mechanism of SMF‐driven pyruvate carboxylation equips the *V. natriegens* model with the option to use it as an alternative anaplerotic carboxylation to the PEP carboxylase, which is also encoded within the *V. natriegens* genome. The presence of both anaplerotic carboxylation enzymes has also been described in bacteria such as *Corynebacterium glutamicum* (Petersen *et al*, 2000). With a 2:1 proton/sodium antiporter stoichiometry, both anaplerotic carboxylations should be equally expensive from an energetic point of view (Fig 5A). The cost of carboxylating pyruvate is equivalent to spending two extracellular sodium ions, which is equivalent to spending four extracellular protons through a 2:1 sodium/proton antiporter stoichiometry. These four extracellular protons can also be used to generate one ATP through ATP synthase. Hence, considering that one ATP is gained by conversion of PEP to pyruvate, the overall cost of both anaplerotic carboxylations should be equivalent. However, the OAD pump has the unique feature of pumping of one proton in the opposite direction to sodium pumping (Di Berardino & Dimroth, 1996). Consequently, the SMF‐driven carboxylation slightly increases the PMF, and the model prefers using the pyruvate carboxylation catalyzed by the Na^+^‐OAD. This was confirmed by simulating growth on glucose, which illustrated that the model preferred using the Na^+^‐OAD in its reverse sense, exploiting the gradient created by the Na^+^‐NQR to drive pyruvate carboxylation.

As the model suggested that Na^+^‐OAD participates in aerobic growth, we hypothesized that deleting this pump would possibly lead to reduced growth. We, therefore, created a knockout (KO) strain for this gene, and a reverted strain harboring the re‐inserted OAD gene. We found that only under high‐salinity conditions, the OAD knockout grew slightly slower and to a lower OD than the WT and OAD reversion strain, while at lower salinity, we observed no difference in growth (Appendix Figs S1 and S2, and Table S2). In the GSMM, knocking out the Na^+^‐OAD and hence forcing the anaplerotic flux through PEP carboxylase *in silico* also slightly decreased growth, by 0.6%. This reduction is relatively small as the model has the option to exploit the 2:1 sodium/proton antiporter to efficiently reallocate the energy stored in the SMF that would otherwise have been used by the Na^+^‐OAD. While we did not find this pump to be significantly differentially expressed between different salinities in the proteomics samples, it was highly expressed in all of them, reaching expression levels in the same order of magnitude as the Na^+^‐translocating NADH:ubiquinone oxidoreductase.

To further investigate the discrepancy between the predicted *in silico* growth rate reduction and the observed growth rate reduction caused by knocking the Na^+^‐OAD, we examined the effect of increased salinity on *V. natriegens'* metabolism in the model. A simple way of doing this is by enforcing an influx of sodium ions across the cytoplasmatic membrane (Fig 5B). The growth rate *V. natriegens* can achieve decreases linearly with sodium influx, as a result of the decreased SMF. At the same time, an enforced sodium influx increases the fraction of the total SMF that is consumed by the Na^+^‐OAD to carboxylate pyruvate, suggesting an increased relative importance of the SMF‐driven carboxylation. This is compatible with the results of the KO experiment which indicated that the Na^+^‐OAD is more important under higher salinity conditions. Alternatively, a further explanation of why the functionality of the Na^+^‐OAD is limited to high salinity environments could be that a low salinity environment may simply not be able to provide the strong sodium gradient that has been described to be required to activate the carboxylation function of the pump (Dimroth & Hilpert, 1984). Taken together, our *in silico* and experimental findings reveal a so far unknown, yet potentially relevant, role of the Na^+^‐OAD in *V. natriegens* metabolism.

### Adaptation to high salinity at the protein level

Finally, we aimed to further investigate *V. natriegens*' halophilic lifestyle by looking at indications of differentially expressed proteins in our proteomics samples. To do this, we generated an overview of significantly differentially expressed proteins between the 0 mM and 300 mM samples according to the one‐way ANOVA test. This overview is provided as Dataset EV9.

Among the proteins that are upregulated under 300 mM NaCl as compared to 0 mM NaCl we found many metabolic enzymes. It is not surprising that diaminobutyrate‐‐2‐oxoglutarate aminotransferase and ectoine synthase from the ectoine synthesis pathway were found to be upregulated at 300 mM, as ectoine is a common osmoprotectant that has been shown to be critical for survival in osmotically stressed *Vibrio* species (Ongagna‐Yhombi & Boyd, 2013). Interestingly, upregulation of biotin synthase suggests an unknown role for biotin at higher salinity.

Many membrane proteins and transporters were upregulated in the 300 mM samples, among which ATP‐binding cassette (ABC) transporters for glycine‐betaine, another osmoprotectant used by *Vibrio* species besides ectoine (Ongagna‐Yhombi & Boyd, 2013). A variety of other ABC transporters were found upregulated, including dicarboxylate, amino acid, and ion transporters. This is not unexpected, as some ABC transporters have been found to be osmosensing enzymes and directly related to osmotic adaptations (Wood, 2015; Bordel *et al*, 2020). Additionally, some of the essential membrane‐bound building blocks of *V. natriegens'* respiratory system were found to be upregulated at 300 mM, such as the Na^+^‐translocating NADH:quinone oxidoreductase, the ATP F0F1 synthase, and cytochrome C. This may indicate that these proteins are better adapted to the transmembrane osmotic forces present at higher salinities, consistent with *V. natriegens'* general preference for such environments.

Finally, a variety of regulatory proteins were found to be upregulated. Among those were transcriptional regulators, heat shock proteins, and universal stress proteins. We found increased levels of anti‐sigma E factor and sigma‐E factor regulatory protein RseB, which suggest a downregulation at higher salinities of sigma E activity, which in *E. coli* is involved in the regulatory response to the accumulation of misfolded or unfolded proteins in the extra cytoplasmatic space (De Las Peñas *et al*, 1997). Cell division protein FtsY was also found to be upregulated. This observation is similar to the increased expression of FtsQ in the halophilic bacterium *Methylomicrobium alcaliphilum* (Bordel *et al*, 2020).

### Allocation of resources during growth on minimal medium

In standard FBA, the value of the substrate uptake rate is the main determinant for the growth rate. This is a limitation of this methodology, which would wrongly suggest that the substrate transporters are the main limiting factor in the process of biomass formation and therefore doubling time. Resource Balance Analysis is a GSMM‐based modeling technique that addresses this limitation of FBA by suggesting that the key element limiting growth rate is the resource allocation of nutrients between all cellular processes (Goelzer & Fromion, 2011). Because fast growth rate is a particularly interesting property of *V. natriegens*, we decided to construct an RBA model based on iLC858.

RBA solves an optimization problem for nutrient allocation between cellular processes and computes optimal abundances of molecular machinery, enzymes, and the corresponding metabolic fluxes. Crucially, RBA ensures that enough metabolic precursors are produced to provide the molecular machinery needed to sustain cellular processes and metabolic fluxes. In addition to metabolic enzymes, the model was constructed to include the major cellular processes of replication, transcription, translation, and protein folding, driven by macromolecular machinery (see materials and methods for details).

Initially, we used the *V. natriegens* RBA model to simulate aerobic growth on glucose. The model was calibrated by estimating apparent catalytic rates for all the enzymes detected in our proteomics dataset using RBApy scripts (Bulović *et al*, 2019). For the remaining enzymes which had not been detected in our proteomics data, a default catalytic rate had to be set. We used a rate of 619,200 h^−1^ for this, based on the average kcat of 172 s^−1^ found for all *E. coli* enzymes studied by Salvy & Hatzimanikatis (2020). The use of this value allowed the simulation to reach growth rates in the range of those reported on minimal medium. Solving the RBA problem yielded a substrate uptake rate of 2.67 g gDW^−1^ h^−1^ and a growth rate of 1.31 h^−1^, approximating the growth rate of 1.4 h^−1^ reported by Hoffart *et al* (2017). The predicted substrate uptake rate 2.67 g gDW^−1^ h^−1^ is lower than the experimentally determined value but also corresponds to a lower growth rate, which might be explained by the more stringent constraints imposed by the RBA model. Furthermore, 75.5% of the *V. natriegens* genes predicted to be expressed by the RBA model at concentrations higher than 10^−6^ mmol.gDW^−1^ were also detected in at least one of the proteomic samples.

Simulating anaerobic growth in the RBA model yielded a substrate uptake rate of 8.03 g gDW^−1^ h^−1^ corresponding to a growth rate of 1.06 h^−1^. This prediction is consistent with a higher substrate uptake rate and lower growth rate under anaerobic conditions reported by Hoffart *et al* (2017), who quantified a substrate uptake rate of 7.81 g gDW^−1^ h^−1^ and a growth rate of 0.92 h^−1^ during anaerobic growth on minimal medium. Additionally, the model predicted anaerobic by‐products formate, acetate, and lactate to be secreted, which were also experimentally detected (Hoffart *et al*, 2017).

Translational machinery, consisting of ribosomal proteins and elongation factors, is a cornerstone of cellular growth, which supplies the cell with proteins to sustain all its processes. Therefore, the allocation of resources towards translational machinery is critical. Under aerobic growth on minimal medium, the RBA model predicted an allocation of 20.5% of total amino acid weight to translational machinery. In the proteomics samples, we found similar fractions of all proteomic counts stemming from translational machinery (Table 2). These values are also similar to the reported 21% of protein capacity allocated to translational machinery in *E. coli* (Li *et al*, 2014). This similarity between *V. natriegens* and *E. coli* protein allocation to translational machinery is interesting, as ribosomal concentration is generally expected to increase with growth rate (Scott & Hwa, 2011), yet the *V. natriegens* growth rate on minimal medium is higher than the growth rate of *E. coli* on minimal medium reported for this 21% proteomic weight fraction of ribosomes (0.74, Li *et al*, 2014).

**Table 2:** Average proteomic weight fraction of ribosomes and corresponding growth rates for the proteomic samples at 300, 540, and 650 mM.

Salinity	Proteomic weight fraction of ribosomes (%)	Corresponding growth rate (h^−1)^
300 mM	21.9 (± 0.6)	1.66 (± 0.07)
540 mM	22.6 (± 0.5)	1.51 (± 0.02)
650 mM	21.6 (± 0.1)	1.16 (± 0.06)

### Conclusions

In this study, we developed and validated the first GSMM for *V. natriegens*. We demonstrated that the model is successful in predicting yields that have been experimentally determined. Furthermore, our model correlates well with experimentally determined carbon source utilization assays. Inaccurate transporter annotations, promiscuous activity of enzymes and low gene expression potentially explain the remaining discrepancies between the model and empirical observations. A model‐wide gene essentiality analysis produced a set of putatively essential genes, which partially overlapped with a high‐throughput CRISPRi gene essentiality study. We anticipate that given the strengths and weaknesses of both approaches, they might serve as complementary approaches to study gene essentiality in *V. natriegens*. Our GSMM also showed good consistency between predicted fluxes and proteomics data which were generated for this study.

*Vibrio natriegens*' most notable property is its capacity to reach exceptional growth speeds. This inspired a comparison between the metabolic networks of *V. natriegens* and the well‐studied model Gram‐negative bacterium *E. coli* (iML1515). When comparing the networks for their theoretical capacities to produce amino acids, we found that *V. natriegens'* Alanine dehydrogenase and L‐alanine:glycoxylate aminotransferase, two genes not found on the *E. coli* genome, provide it with a metabolic advantage over *E. coli* for the production of the amino acid glycine. We did not detect high levels of expression of these genes in our proteomics samples of growth on minimal medium but found evidence of high transcription of these genes on MOPS medium in a ribosomal profiling dataset (Lalanne *et al*, 2018), suggesting that this potential metabolic advantage may only be used under certain conditions.

However, when comparing iLC858's capacity to produce an array of biomass precursors to the *E. coli* networks iJO1366 and iML1515, we found no overall significant advantage for the *V. natriegens* network. *V. natriegens* does, therefore, not seem to hold an obvious intrinsic advantage in its metabolic pathways leading to biomass precursors when compared to *E. coli* that could help explain its rapid growth. Hence, from a genome‐scale metabolic modeling point of view, a major requirement and determinant for high growth rates in *V. natriegens* still seems to be its high substrate uptake rate, as was also pointed out by Hoffart *et al* (2017).

*Vibrio natriegens'* halophilic nature requires major physiological adaptation to high salinity environments. This manifests itself in *V. natriegens'* respiratory chain which is crucially different to the one in *E. coli* and represented a key target for curation in iLC858. *V. natriegens'* primary respiratory pump is the Na^+^‐translocating NADH:quinone oxidoreductase (Na^+^‐NQR), a unique enzyme whose central role has been studied in *V. cholerae* and which has no equivalent on the *E. coli* genome (Steuber *et al*, 2015). Instead, *E. coli* encodes a proton‐pumping NADH:ubiquinone oxidoreductase that is missing in the genome of *Vibrio* spp. (Minato *et al*, 2014). The central role of the Na^+^‐NQR was also suggested by the GSMM simulations as it was predicted to be the highest flux *V. natriegens* reaction with no equivalent in *E. coli*. In the proteomics samples, we detected significant upregulation at 300 mM of the Na^+^‐NQR, as well as the ATP synthase. This could indicate an adaptation of the respiratory chain *V. natriegens'* to its halophilic lifestyle.

A Na^+^‐translocating oxaloacetate decarboxylase (Na^+^‐OAD) was highlighted as another functional membrane pump interacting with *V. natriegens'* sodium gradient. The role of this pump was so far unknown. Simulations with the GSMM suggested that this pump might work as a gradient‐driven pyruvate carboxylation and, therefore, an alternative anaplerotic carboxylation. We detected expression of this pump in our proteomics data, in high levels in the same order of magnitude as the Na^+^‐NQR. Further experimental work with a knockout strain for this pump supported the functionality of the Na^+^‐OAD pump during aerobic growth. To our knowledge, this is the first demonstration of the functionality of this enzyme during aerobic growth in bacteria. These observations suggest that this enzyme plays a role during *V. natriegens* growth that has no equivalent in *E. coli*. Moreover, they may provide insights into the lifestyle of the important human pathogen *V. cholerae*, as it also possesses a homologous Na^+^‐OAD that is decoupled from the citrate fermentation operon (Dahinden *et al*, 2005).

Production of proteins by translational machinery essentially limits the rate at which a cell can accumulate biomass to grow and proliferate. The correlation of cellular resource allocation to ribosomes during cellular growth with growth rate has been studied in *E. coli* and has furthermore been accepted as a universal growth law (Jin *et al*, 2012; Scott *et al*, 2014). Aiyar *et al* (2002) found that total RNA/protein ratios in *V. natriegens* increase with growth rate but did not experimentally test this for specifically ribosomes (Aiyar *et al*, 2002). Furthermore, the high number of 11 rRNA operons on the *V. natriegens* genome suggests a connection to its capacity for rapid growth. We, therefore, sought to compare the allocation of total amino acid weight to translational machinery in *E. coli* and *V. natriegens*. In our proteomics samples of *V. natriegens* growth on glucose minimal medium with 300 mM NaCl, we found an allocation of 22.6% amino acid cellular dry weight allocated to translational machinery in *V. natriegens* corresponding to a growth rate of 1.6 h^−1^. This was in good agreement with the RBA model simulations which predicted a growth rate of 1.4 h^−1^ for a translational machinery amino acid allocation of 20.5%. Yet, in *E. coli* amino acid allocation to translational machinery of 21% during growth on glucose minimal medium has been reported to sustain a growth rate of only 0.74 h^−1^. It, therefore, seems that for the same amount of ribosomal resource allocation *V. natriegens* manages to achieve higher growth rates. Moreover, we did not find a significant correlation between ribosomal resource allocation and different growth rates across proteomics samples corresponding to three different salinities, as opposed to the expected increase in ribosomal resource allocation with increasing growth rates.

Genome‐scale models have many uses in metabolic engineering such as prediction of maximum theoretical yields, identification of metabolic bottlenecks and identification of knockout targets for increasing product yields. As such, we anticipate that our GSMM as well as our RBA model will serve as useful tools to guide metabolic engineering efforts in *V. natriegens*, as well as further studies into the metabolic intricacies of this fast‐growing bacterium.

## Materials and Methods

### Construction of the model

The genome of *V. natriegens* strain ATCC 14048 (NCBI accession CP009977.1 and CP009978.1) was annotated using RAST (Overbeek *et al*, 2014). An automated draft construction of the model was generated using the SEED server (Overbeek *et al*, 2005). We did not use the gapfilling option provided by SEED. Instead, missing reactions were manually added to the network based on knowledge retrieved from the RAST annotation, NCBI BLAST (Altschul *et al*, 1990) and the KEGG (Kanehisa *et al*, 2012) database. Model curation was performed using COBRApy (Ebrahim *et al*, 2013). Our GSMM, named iLC858, is available in SBML format from https://github.com/LucasCoppens/Modelling_Vibrio_natriegens.

### Constraint‐based growth simulations

Flux balance analysis was used to calculate biomass yields and growth rates for *in silico* substrate assays and gene essentiality analysis. Biomass formation was set as the objective, and the carbon substrate uptake rates were used as constraints. In addition, lower bounds on CO_2_ production and acetate secretion were set in order to simulate exponential growth on minimal medium. ATP maintenance was not considered as a separate constraint as it was included in the biomass equation. All simulations were carried out using the COBRApy package (Ebrahim *et al*, 2013).

### Experimental carbon substrate assays

*Vibrio natriegens* ATCC 14048 was submitted to BioLog (Hayward, CA, USA) for Phenotype MicroArrays (PMs) for Microbial Cells™ analysis on the OmniLog System®. Cells were grown on BioLog Universal Growth media with sheep's blood at 30°C overnight. They were then added into the wells of the plates, which were run according to a proprietary protocol for running Gram‐negative bacteria. The base test media for the metabolic test plates is proprietary, but it is a minimal media composed mostly of salt and buffers. For more information, see the BioLog website (https://www.biolog.com/products‐portfolio‐overview/phenotype‐microarrays‐for‐microbial‐cells/) and reference papers Bochner *et al* (2001) and Zhou *et al* (2003).

### *In silico* carbon substrate assays

Codes corresponding to carbon substrates were manually retrieved from the modelSEED database (Overbeek *et al*, 2005). *In silico* carbon substrate assays were only performed on the carbon sources that were represented in the model as extracellular or intracellular metabolites. Correspondingly, the ability of *V. natriegens* to grow on substrates *in silico* was evaluated using an extracellular and intracellular sink. The outcomes of both the *in silico* and experimental growth assays were classified as “viable” or “not viable,” with viable being the models ability to produce biomass solely from the substrate in question as carbon source.

### Substrate biomass yields

For the determination of biomass yields, *V. natriegens* was grown in 3 mL modified M9 minimal medium (11.28 g 5X M9 salts [Sigma], 6.78 g Na_2_HPO_4_, 3 g KH_2_PO_4_, 2 mM MgSO_4_, 0.1 mM CaCl_2_, 25 μM FeCl_3_, 300 mM NaCl, pH 7.4) with 10 g l^−1^ of carbon substrate, at 700 rpm. Fully grown cultures were centrifuged and dried to measure dry weight. Yield was then calculated as the final dry weight divided by the initial carbon substrate weight.

### *In silico* gene essentiality

Gene knockouts were simulated by applying the COBRApy built‐in knockout function to genes (Ebrahim *et al*, 2013). FBA was subsequently performed on these *in silico* knockout strains, using biomass formation as an objective. Correspondingly, genes where knockouts lead to infeasible FBA, or a growth rate reduced below 90% of nonimpaired growth were classified as “essential.”

### Generation of *V. natriegens* KO and *in situ* complement strains

The *V. natriegens* OAD knockout strain (OAD KO) was generated using the MuGENT protocol (Dalia *et al*, 2017). This was used to replace the whole OAD gene with the chloramphenicol (Cmp) cassette in the genome. *V. natriegens* 14048 RifR (spontaneous Rifampicin mutant) with the pMMB67EH‐‐tfox(Vc) AmpR plasmid was used as the base/wild‐type strain for genome modification.

First, fusion PCR was used to generate a 5.2‐kb linear fragment in which the Cmp cassette was fused with 2‐kb flanking regions of the OAD gene. The Phusion Flash Master Mix (ThermoFisher) was used. As per manufacturer's instructions, the three fragments to be fused were first separately amplified. The cassette was amplified using the primers Cm4OAD‐F (aacatcctgcacctgtcaGAGCGATTGTGTAGGCTG) and Cm4OAD‐R (gcgagtatgttacccacaTGGGAATTAGCCATGGTCC), the upstream fragment was amplified using primers OADup‐F (CGTTGCTTCAGGTAACTACTAA) and OADup‐R (cagcctacacaatcgctcTGACAGGTGCAGGATGTT), and the downstream fragment was amplified using OADdn‐F (ggaccatggctaattcccaTGTGGGTAACATACTCGC) and OADdn‐R (GGATATAACGCTTCTGACATG). These were run as 20‐μl reactions, gel‐verified, and purified using a Zymo DNA Clean and Concentrate Kit as per manufacturer's instructions. To perform the fusion reaction, 15 μl of Master Mix, 2 μl of the Cmp cassette, and 1 μl of each of the flanking cassettes were added to a 30‐μl reaction (total volume). The following program was run: 98°C for 1 min, then 11 cycles of: 98°C for 1 s, 75°C for 1 s, Ta ‐ ramped down to 72?C at −0.1°C/s from 75°C, hold for 30 s at 72°C for 10 min. For the purification reaction, 5 μl of the above reaction was taken and added to 50 μl of Master Mix, 0.5 μM each of nested primers (OADCm‐F: AATCGGCATCGGTTAAGT; OADCm‐R: CTTAACCGTAGAACGTTGAAT) in a 100 μl reaction. A 2 step PCR was run, as per the manufacturer's protocol. The purified band was gel verified and purified using the Zymo DNA Clean and Concentrate Kit, and then used in the MuGENT protocol (Dalia *et al*, 2017). In the MuGENT protocol, 50 ng of total purified fused fragment was added to the cells. To check for cassette insertion into the genome, the primers CmR‐ck‐F: ACTGACTGAAATGCCTCAA and OAD‐Cmr‐pur‐R: GACGGTTATCGTAACGTTGA were used in a boiled‐colony PCR using Taq DNA Polymerase (New England Biolabs) as per the manufacturer's protocol. The size and sequencing of the insert, as compared to a control, confirmed deletion of OAD and insertion of the cassette into the genome, as did chloramphenicol resistance.

The confirmed OAD KO strain was the complemented *in situ* back with the OAD gene. To do this, the MUGENT protocol was run as before, but with two tDNA cassettes: one cassette was created with the primers OADup‐F (CGTTGCTTCAGGTAACTACTAA) and OADdn‐R (GGATATAACGCTTCTGACATG) to amplify the OAD gene and up and downstream regions out of the *V. natriegens* genome. A second cassette was created by replacing the DNS gene in *V. natriegens* with the ampicillin resistance cassette. The cassette was created as following: primers for region upstream of DNS: Dns‐3 kb‐F (ctaacatggctaagcacctg) and DNS4AmpR‐R (tatttcctattgagacttaattgaactgagg); Primers for ampR cassette: AmpR‐F(tcaataggaaatattagacgtcaggtggcact) and AmpR‐R (aaagattagcgattgagtaaacttggtctgacagttacc); Primers for region downstream of DNS: DNS4AmpR‐F (ccaagtttactcaatcgctaatctttctgtttgagg) and DNS‐3 kb‐R (actggtaagccataacgacc). MUGENT with the addition of 100 ng of the DNS:Amp cassette and 250 ng of the OAD complement cassette was preformed, and cells were selected on ampicillin. The colonies that grew were then patched on Cmp, and those which did not grow on Cmp were confirmed for OAD *in situ* complementation and Amp insertion at DNS by colony PCR and sequencing. MUGENT was also performed on the *V. natriegens* RifR and RifR OAD KO strains with the DNS:AmpR cassette only to create appropriate controls.

### OAD KO, complement, and WT growth assays

The indicated *V. natriegens* strains were inoculated and grown overnight from glycerol stocks at 30°C in modified minimal medium (11.28 g 5X M9 salts (Sigma), 6.78 g Na_2_HPO_4_, 3 g KH_2_PO_4_, 2 mM MgSO_4_, 0.1 mM CaCl_2_, 25 μM FeCl_3_, indicated amount of NaCl, and 0.4% glucose, pH 7.4). The following morning, cells were diluted 1:200 in the same media and dispensed in triplicate into the wells of a BioScreen C Analyzer 100‐well honeycomb plate (Growth Curves USA, Piscataway, NJ, USA), 150 μl per well. Growth was measured at OD_600_, with measurements taken every 15 min. for 24 h. All chemicals, including carbon sources, were from Sigma‐Aldrich (Sigma‐Aldrich, St. Louis, MO, USA) or Fisher Scientific (Thermo Fisher Scientific, Waltham, MA, USA).

### Proteomic analyses

*Vibrio natriegens* (ATCC 14048) was grown in a modified M9 minimal medium (11.28 g 5X M9 salts [Sigma], 6.78 g Na_2_HPO_4_, 3 g KH_2_PO_4_, 2 mM MgSO_4_, 0.1 mM CaCl_2_, 25 μM FeCl_3_, pH 7.4) supplemented with glucose and NaCl as indicated below. Three different colonies of the expected morphology were used to inoculate each test tube containing modified M9 minimal medium with 0.4% (w/v) glucose and 0 mM NaCl and grown for 16 h at 36.5°C and 250 rpm in an orbital shaker. Based on the density of the overnight cultures, using a conversion factor of 1.75 × 10^8^ CFU ml^−1^ OD_600_^−1^, the appropriate volumes containing ∼ 10^5^ cells were transferred to flasks containing prewarmed modified M9 minimal medium with 1% (w/v) glucose and either 0, 300, 540, or 650 mM NaCl. The flasks were incubated at 36.5°C with continuous shaking and OD_600_ measurements were monitored, and samples were collected during early log, mid‐log, and stationery phases of growth. Aliquots for proteomic analysis (2 ml) were taken directly from the flasks at the specified growth points and pelleted at 5,000 *g* for 1 min. at 20°C.

Cell pellets were taken from −80°C storage and placed on ice. Lysis buffer (10% n‐propanol in 50 mM ammonium bicarbonate) was added and volumes adjusted based on optical density measurements (as detailed in Dataset EV10). Pressure cycling technology (PCT) was used for cell lysis and proteolytic digestion with trypsin (Schultzhaus *et al*, 2019). For each sampling point, 100‐μl suspensions were transferred to PCT tubes (PressureBiosciences Inc., South Easton, MA) for lysis. Samples were lysed under pressure in the barocycler (HUB 440 High Pressure Generator, PressureBiosciences Inc.) with pressure cycling on for 20 s and off for 10 s at 30°C for 60 cycles. Lysate (50 μl) was transferred to a new PCT tubes for digestion along with 45 μl of lysis buffer and 5uL of modified sequencing grade trypsin stock solution (item V5111, Promega, Madison, WI, USA; stock concentration 0.02 μg/ml). Extracted proteins from lysed cells were digested under pressure in the barocycler with pressure cycling for 50 s on, 10 s off, at 37°C for 60 cycles. Resulting digests were dried in speed‐vac (Thermo Savant, Ashville, NC, USA) and stored at −20°C prior to analysis by liquid chromatography tandem mass spectrometry (LC–MS/MS).

Immediately prior to LC–MS/MS analysis, digests were reconstituted in 50 μl of 0.1% formic acid in water (solvent A). The LC–MS/MS system consisted of an Ultimate U3000 LC coupled with an Orbitrap Fusion Lumos mass spectrometer (Thermo Scientific, Waltham, MA, USA) via a Nanospray Flex Ion Source. The U3000 system was configured for nano‐flow separations using an autosampler, loading pump, and analytical pump. The autosampler loaded samples onto a trap column (PepMap 100, C18, 300 μm ID × 5 mm, 5 μm, 100 Å) via loading pump [10 μl/min flow rate and 98% solvent A, 2% solvent B (0.1% formic acid in acetonitrile)]. The analytical pump (flow rate 300 nl/min) was used to elute peptides from the trap onto an analytical column (Acclaim PepMap RSLC, 75 μm ID × 150 mm, C18, 2 μm, 100 Å) and into the mass spectrometer. Mass spectra were acquired on a Fusion Lumos Orbitrap mass spectrometer in data‐dependent mode with 3 s cycle times. A survey scan range of 400–1,600 Da was acquired on the Orbitrap detector (resolution 120 K). The maximum injection time was 50 ms and automatic gain control (AGC) target was set to standard. Ion intensities > 5.03 with predicted charges of 2+ to 7+ were fragmented using higher energy collisional dissociation (HCD). Dynamic exclusion was enabled for a duration of 30 s for subsequent MS/MS submissions. The MS/MS detector was set to Ion Trap rapid scan with maximum injection time 35 ms and AGC target set to standard.

Mass spectrometry data were extracted, converted, and searched by Mascot (v. 2.6.2, Matrix Science LTD, London, UK) via in‐house scripting (Hervey *et al*, 2009). Mascot matched tandem mass spectra to a sequence database consisting of the *V. natriegens* ATCC 14048 predicted proteome and 190 sequences representing common contaminants, proteolytic enzymes, and mass standards (e.g., trypsin, keratin, etc.). Variable modifications for the search included oxidation of methionine and deamidation of glutamine and asparagine. Proteolytic cleavage search parameters used trypsin with > 3 missed cleavages. The precursor ion tolerance was set to +50 ppm and fragment ion to +0.6 Da. Scaffold (v. 4.8.9, Proteome Software Inc., Portland, OR, USA) was used to validate MS/MS based peptide and protein identifications. Following Scaffold delta‐mass correction, identifications were retained at >80.0% probability and > 95.0% probability at the peptide (Keller *et al*, 2002) and protein levels (Nesvizhskii *et al*, 2003). Proteins that contained similar peptides and could not be differentiated based on MS/MS analysis alone were grouped to satisfy the principles of parsimony. Spectrum counts were normalized using the standard Scaffold normalization procedure which normalizes the counts for each detected protein in a sample with respect to the total protein counts per analyzed sample to allow cross‐sample comparison. Analysis of Variance (ANOVA) without correction for multiple testing was performed on the normalized weighted mass spectra assigned to the proteins for relative quantification. Primary mass spectrometry (MS) data are available in the Mass Spectrometry Interactive Virtual Environment (MassIVE) via accession MSV000090243 at the following URL: https://massive.ucsd.edu/ProteoSAFe/dataset.jsp?task=a722a4c609204beda2bc6ac2b9575f16.

### Resource balance analysis model

The RBApy package was used to create the RBA model (Bulović *et al*, 2019). Additionally, the extension of this package by Michael Jahn was used to include the processes of transcription and replication and their corresponding machineries in the model (Jahn *et al*, 2021). The model is available at https://github.com/LucasCoppens/Modelling_Vibrio_natriegens. The RBA model builds on the GSMM iLC858. Besides the GSMM, sequences and compositions for ribosomes, chaperones, DNA polymerase, and RNA polymerase were manually retrieved from the annotated genome of *V. natriegens* ATCC 14048 (NCBI accession CP009977.1 and CP009978.1). Rates for these processes were taken from Jahn *et al* (2021), in which values adopted from *E. coli* were used. Information for enzymes like sequence subunit stoichiometry and cofactors were automatically retrieved from the Uniprot database. As Uniprot only partially covered the proteins included in the model, we manually extended the Uniprot file to include all the required information.

Macrocomponent production targets were set to match the macrocomponent production targets of the *E. coli* RBA model based on their similarities as fast‐growing Gram‐negative bacteria (Bulović *et al*, 2019). The biomass contributions of two phospholipids (pe160 and pe161), bactoprenyl diphosphate (udcpdp), kdo2‐lipid IVA (kdo2lipid4), and biotin (btn), which were represented as metabolites in both models, were adopted from the *E. coli* to the *V. natriegens* model. The murein precursor uaagmda found in the Vnat model was used to represent biomass contributions to the peptidoglycan layer. Additionally, 0.1 g g^−1^ poly‐β‐hydroxybutyrate (PHB) and 0.03 g g^−1^ ectoine production targets were taken from literature on *Vibrio* (Chien *et al*, 2007; Van Thuoc *et al*, 2021).

Finally, the model was calibrated estimating apparent catalytic rates for all the enzymes detected in our proteomics dataset. For this, we followed the procedure proposed by Jahn *et al* (2021), which is based on the generation of metabolic flux boundaries through flux sampling using FBA and enzyme abundance as determined by proteomics. A protein weight fraction of 47% dry weight determined by Long *et al* (2017) was used to scale the relative protein abundances as determined by proteomics to concentrations per gram of dry weight. The RBApy built‐in script was used for the estimation of apparent catalytic rates. RBApy built‐in scripts were also used to determine the proteome fraction per cellular compartment and proteome nonenzymatic protein fraction per compartment as a function of growth rate.

For RBA growth simulations, an acetate production rate was enforced at a value of 0.42 g gDW^−1^, as reported by Hoffart *et al* (2017).

### Proteomics differential expression analysis

The Python package scipy was used to perform one‐way ANOVAs between the 0 mM and 300 mM NaCl proteomics samples.

## Author contributions

**Lucas Coppens:** Data curation; formal analysis; investigation; visualization; methodology; writing – original draft. **Tanya Tschirhart:** Data curation; formal analysis; writing – review and editing. **Dagmar H Leary:** Data curation; formal analysis. **Sophie M Colston:** Data curation; formal analysis. **Jaimee R Compton:** Data curation; formal analysis. **William Judson Hervey:** Data curation; formal analysis. **Karl L Dana:** Data curation; formal analysis. **Gary J Vora:** Resources; methodology; writing – review and editing. **Sergio Bordel:** Data curation; formal analysis; investigation; methodology. **Rodrigo Ledesma‐Amaro:** Conceptualization; resources; data curation; formal analysis; supervision; funding acquisition; investigation; writing – original draft; project administration; writing – review and editing.

## Disclosure and competing interests statement

The authors declare that they have no conflict of interest.

## Supporting information



Appendix S1

Dataset EV1

Dataset EV2

Dataset EV3

Dataset EV4

Dataset EV5

Dataset EV6

Dataset EV7

Dataset EV8

Dataset EV9

Dataset EV10

## Data Availability

The GSMM iLC858 as well as the Resource Balance Analysis model produced in this study are available at: https://github.com/LucasCoppens/Modelling_Vibrio_natriegens. Primary mass spectrometry (MS) data have been deposited in the Mass Spectrometry Interactive Virtual Environment (MassIVE) and assigned accession MSV000091219. This complete MS data submission has been mirrored via the PRoteomics IDEntification database (PRIDE), assigned the PRIDE accession PXD039932, and is available across proteomeXchange: http://proteomecentral.proteomexchange.org/cgi/GetDataset?ID=PXD039932.
