# Supplementary material for: Vibrio natriegens genome‐scale modeling reveals insights into halophilic adaptations and resource allocation
Source: Mol Syst Biol. 2023 Feb 27;19(4):e10523. doi: 10.15252/msb.202110523 (PMC10090949; doi:10.15252/msb.202110523)
Supplement: Supplementary file 1 — Appendix S1 [file MSB-19-e10523-s008.pdf]

# Appendix

**Table of Content**


1. **Appendix Table S1……1**
2. **Appendix Table S2……1**
3. **Appendix Figure S1……2**
4. **Appendix Figure S2……2**


| | | iLC858 | |
|---|---|---|---|
| | 858 | Essential 182 | Non-essential 676 |
| Lee et al. (2016) | Essential 171 | 83 | 88 |
| | Non-essential 687 | 99 | 588 |

| Measure | Value |
|---|---|
| Sensitivity | 0.4854 |
| Specificity | 0.8559 |
| Precision | 0.456 |
| Negative Predictive Value | 0.8698 |
| False Positive Rate | 0.1441 |
| False Discovery Rate | 0.544 |
| False Negative Rate | 0.5146 |
| Accuracy | 0.7821 |
| F1 Score | 0.4703 |
| Matthews Correlation Coefficient | 0.3335 |

**Appendix Table S1. Confusion matrix comparing the gene essentiality data between iLC858 gene essentiality predictions and the gene essentiality study performed by Lee et al. (2016)**

| 0 mM | | 300 mM | |
|---|---|---|---|
| **Sample** | **Timepoint at which $OD_{600}$ = 1.0** | **Sample** | **Timepoint at which $OD_{600}$ = 1.0** |
| WT 1 | 9.5 | WT 1 | 8.2 |
| WT 2 | 9.5 | WT 2 | 8.3 |
| WT 3 | 9.5 | WT 3 | 8.3 |
| OAD KO 1 | 9.8 | OAD KO 1 | 9 |
| OAD KO 2 | 9.8 | OAD KO 2 | 8.8 |
| OAD KO 3 | 9.8 | OAD KO 3 | 8.8 |
| OAD reverted 1 | 10 | OAD reverted 1 | 8.2 |
| OAD reverted 2 | 9.8 | OAD reverted 2 | 8.2 |
| OAD reverted 3 | 9.8 | OAD reverted 3 | 3 |

**Appendix Table S2. Time it takes for the WT, OAD KO and OAD reverted strains cultures in Figure S2 to reach $OD_{600}$ = 1**

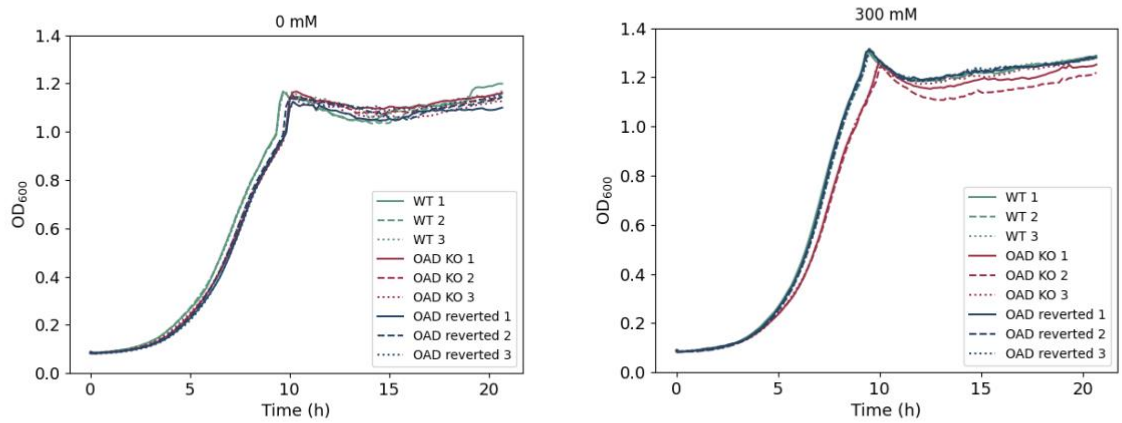


**Appendix Figure S1. Growth curves of the individual replicates of the WT, OAD KO and OAD reverted strains on 0 mM and 300 mM**

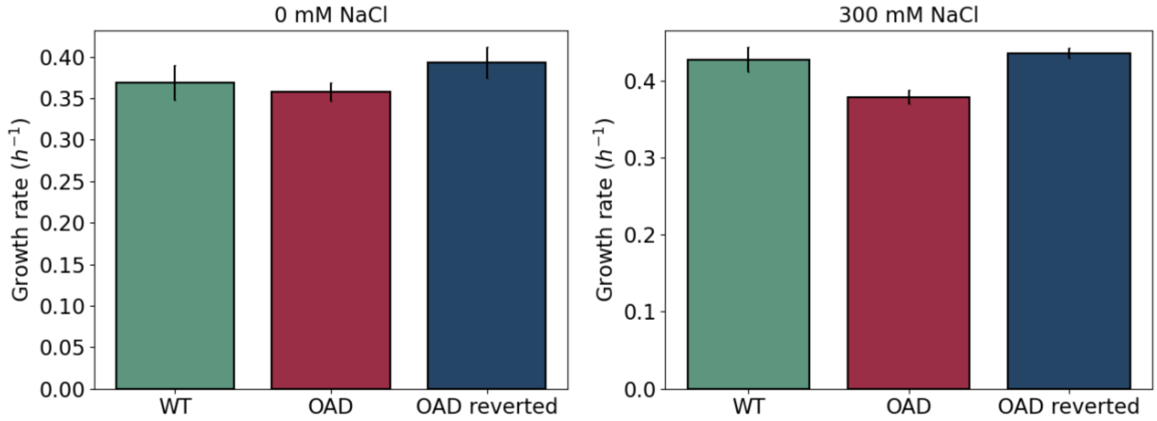


**Appendix Figure S2. Mean growth rates of the WT, OAD KO and OAD reverted strains cultures from Figure S2 at OD = 0.3**
