# Supplementary material for: Vibrio natriegens genome‐scale modeling reveals insights into halophilic adaptations and resource allocation
Source: Mol Syst Biol. 2023 Feb 27;19(4):e10523. doi: 10.15252/msb.202110523 (PMC10090949; doi:10.15252/msb.202110523)
Supplement: Supplementary file 12 [file MSB-19-e10523-s003.pdf]

molecular systems biology

Vibrio natriegens genome-scale modelling reveals halophilic adaptations and resource allocation

Lucas Coppens, Tanya Tschirhart, Dagmar Leary, Sophie Colston, Jaimee Compton, William Judson IV Hervey, Karl Dana, Gary Vora, Sergio Bordel, and Rodrigo Ledesma-Amaro
DOI: 10.15252/msb.202110523

Corresponding author(s): Rodrigo Ledesma-Amaro (r.ledesma-amaro@imperial.ac.uk)

Review Timeline:

Submission Date:	22nd Jun 21
Editorial Decision:	6th Aug 21
Revision Received:	21st Aug 22
Editorial Decision:	27th Sep 22
Appeal Received:	19th Oct 22
Editorial Decision:	21st Oct 22
Revision Received:	17th Jan 23
Editorial Decision:	31st Jan 23
Revision Received:	7th Feb 23
Accepted:	9th Feb 23

Editor: Jingyi Hou

Transaction Report:

(Note: With the exception of the correction of typographical or spelling errors that could be a source of ambiguity, letters and reports are not edited. Depending on transfer agreements, referee reports obtained elsewhere may or may not be included in this compilation. Referee reports are anonymous unless the Referee chooses to sign their reports.)

1st Editorial Decision 6th Aug 2021

Thank you for submitting your work to Molecular Systems Biology. We have now heard back from two of the three reviewers who agreed to evaluate your manuscript. Unfortunately, after a series of reminders, we did not manage to obtain a report from Reviewer #1. In the interest of time, I prefer to make a decision now rather than further delaying the process. As you will see below, the reviewers raise substantial concerns about your work, which unfortunately preclude its publication in Molecular Systems Biology.

The reviewers acknowledge that the construction of a genome-scale metabolic model for Vibrio natrieagens is of potential interest. While Reviewer #3 (whose expertise is in synthetic biology and V. natriegens) is more supportive and does not raise major issues about the model, Reviewer #2 (whose expertise is in metabolic modeling and microbiology) raises significant concerns with regard to the quality control and validation of the model, the lack of detailed comparison to other Vibrio metabolic models, and insufficient experimental validations of several hypotheses generated by the model. In particular, Reviewer #2 rated the "conceptual novelty", "Adequacy of methods analysis and validation", and "suitability for publication" as "Low/Unaccepted". During our pre-decision cross-commenting process (in which the reviewers are given a chance to make additional comments, including on each other's reports), Reviewer #2 thinks that the study remains at a preliminary stage and as it stands the overall biological novelty provided remains limited. The reviewer did make constructive suggestions regarding further experimental and computational analyses that should be performed to better support the model and the main conclusions. However, performing these analyses would go significantly beyond the scope of major revision and would involve substantial further analyses with unclear outcomes. As such, at this point, we see no choice but to return the manuscript with the message that we cannot offer to publish it.

Nevertheless, we would not be opposed to considering a substantially revised and extended manuscript based on this work, provided that the issues raised by the reviewers can be convincingly addressed. Some of the more essential issues that would need to be thoroughly addressed include:

- Reviewer #2's concerns regarding the quality control and validation of the model must be addressed.
- A comparison between the current model and other Vibiro models needs to be performed and reported.
- Additional experimental validations would be needed to fully support the model predictions, as suggested by Reviewer #2.

All other concerns need to be addressed as well. Of course, I understand if in light of the substantial revisions required, you prefer to submit your study elsewhere.

Reviewer #2:

The manuscript by Coppens and colleagues describes the reconstruction of a genome-scale metabolic model for the fast-growing and biotechnologically relevant organism Vibrio natriegens. The authors indicate that such model is a necessary computational tool that will allow further development of the strain as a chassis for industrial applications. The authors suggest an important role for the sodium-dependent oxaloacetate decarboxylase pump, and create a mutant to perform laboratory experiments under low and high salinity. While there is a clear value of a genome-scale metabolic model reconstruction for the biotechnologically relevant organism V. natriegens, comparison to other relevant models and limited. Quality control and validation of the model with experimental data are also limited, which makes it more difficult to appreciate the conclusions of this work. The overall novelty in terms of methodology is low and while the hypotheses generated with the model could be interesting, they have yet to be fully validated experimentally.

MAJOR CONCERNS:

--Memote is a useful tool for quality control and to ensure that the best practices already used in software development are adopted. The draft reconstruction was automatically built with the SEED server and manually curated (potentially flawed in quality, ensure compatibility with other model identifiers). The number of reactions that were gap-filled by SEED would be important to present here. It would also be important to clearly indicate if Memote was run on this metabolic model reconstruction. Also, metabolic model names usually include the number of genes and not the number of metabolites as presented here.

--There is no detailed comparison to other Vibrio metabolic models. The authors highlight the greater number of metabolites in their reconstruction but do not show more substantial comparisons with related Vibrio models. It would be important to explain the main differences between these Vibrio models in addition to E. coli (the biomass composition could also be compared to iML1515 instead of iJO166). What about the number of shared reactions between these models? Was the ID mapping performed? Flux variability analysis? Shadow prices of the models?

--Additional experimental validations would be needed to fully support the model. For example, Page 4, line 6: "Even under aerobic conditions, V. natriegens secretes part of its substrate as acetate, indicating a mixed respiratory-fermentative growth phenotype". This would be an interesting experimental validation to perform that is not excessively complicated. On the same idea, the fluxes observed in the model could be compared to the expression of the corresponding enzymes using transcriptomics or ribosome profiling datasets (see this dataset as an example: https://www.ncbi.nlm.nih.gov/geo/query/acc.cgi?acc=GSM2500126). This would provide another metric to evaluate the model compared to experimental data. Transposon mutagenesis, although not absolutely required, would also be an alternative or complement the CRISPRi data.

The quality of the predictions for gene essentiality should be delivered by calculating accuracy and precision. The Matthews correlation coefficient should also be considered as a metric for the quality of predictions. As currently presented, it is difficult to clearly obtain these metrics.

--Page 5, line 28: "The model predicts similar growth rates (~1.6 h-1) on glucose, galactose, rhamnose, maltose, glycerol, fructose, sucrose, glucosamine and N-acetylglucosamine. This demonstrates that the enzymes required to utilize these carbon sources at similar efficiencies as glucose are encoded within the V. natriegens genome". This indicates that the genes catalyzing these reactions were found in the genome and that the reactions responsible for their degradation have a similar stoichiometry. The bounds on the model were not changed and the exchange flux (not mentioned) was probably maintained at 3.9 like for glucose. A kinetic model and catalytic constants would be required to make such a claim.

MINOR COMMENTS:

-I would suggest using page and/or line numbers in future manuscript submissions to facilitate the review process.

-Page 7, line 1: "The vast majority (92.8%) of reactions in our model, not counting passive transmembrane transport reactions or metabolite sink reactions, are associated with at least one known coding sequence in the V. natriegens genome". Details about this should be presented earlier in the model reconstruction section:

--Figure 1. Please cite directly on the figure the source for the data that was used to validate the model. As currently represented, this suggests that the data was generated exclusively for this paper.

--Figure 2. a) Choosing a color other than blue would accentuate the contrast between the grey lines and the dots. Reaction names (human-readable) would be easier to understand instead of SEED identifiers. Units should be represented on the Y-axis. Coloring reactions by subsystem would facilitate the interpretation of this graph.
b) How do model predictions compare with experimental observations here? As currently represented it is not possible to get this information.

--Figure 3. b) and c) The quality of the predictions should be delivered by calculating accuracy and precision. The Matthews correlation coefficient should also be considered as a metric for the quality of predictions.

Reviewer #3:

The authors construct the first genome-scale metabolic model for the fast-growing bacterium Vibrio natriegens using available genome sequence data. They validate the curated model by showing impressive agreement between the predicted growth values and metabolic fluxes and the corresponding experimental results from Hoffart et al and Long et al., and also make comparisons with gene essentiality data from Lee et al. and the authors' own carbon source utilization results, showing very reasonable levels of agreement in both cases, including plausible suggestions about sources of disagreement. They take initial steps to experimentally test an interesting functional prediction of their model, that an oxaloacetate decarboxylase (Na+-OAD pump) may play an important role in aerobic growth in high salinity, in a function supported by previous biochemical work. Overall, the authors provide a compelling description and demonstration of the value of GSMM building and how it could influence experimental engineering or evolution efforts. The methods used do not seem technically innovative but they are rigorously applied and the results represent a significant contribution to understanding and manipulating this emerging model organism. The paper should be of interest to those working in Vibrio biology, metabolic engineering in general (since it highlights and enables the usefulness of V natriegens as a new chassis), and metabolic biochemistry. I strongly recommend this paper for publication, subject to the following revisions.

Major points - straightforward but important to correct:
The authors say: "It is possible then that the ED pathway may be activated as an alternative to glycolysis depending on the carbon source, as is the case when E. coli is grown on gluconate (Eisenberg and Dobrogosz, 1967), or generally depending on V. natriegens' energy supply (Flamholz et al., 2013)"
Eagon and Wang, J Bacteriol 1961, give direct experimental evidence that ED enzymes are activated by growth on gluconate! So the speculation here seems to be correct, and the reference should be cited and discussed.

Although I am not enough of a proteomics specialist to say for sure, the data underlying the claim that the Na+-OAD is differentially expressed in low and high salt conditions seems to need more support, qualification or explanation. The 5x differential expression figure seems to come from raw counts of peptides but the methods don't describe measures to normalize for total protein content of the samples or to place Na+-OAD levels in the context of overall protein expression. The differential expression claim is not unreasonable given the knockout/growth data and the modeling results, which are persuasive, so I might suggest discussing it more tentatively in the text, or, if the proteomics methods used are indeed standard and reasonable in the context of the technique, this could be spelled out in the text and perhaps also the methods, to support the idea that differences in peptide count are a good proxy for differential expression.

The Figure 4b caption and figure don't make clear that the right-hand panel is under high salinity and the left-hand panel low salinity. The figure panels should have separate captions or be labeled clearly on the figure itself, and the caption should give the salinity used. The methods seem to imply that this would be given in the figure/caption and it's missing; it should be in both places. The meaning of the -1, -2 and -3 conditions should be spelled out in the caption, not just in the methods (assuming these are the triplicate wells from a single experiment). Ideally, replicates from a single experiment would be averaged and the entire experiment repeated on {greater than or equal to}3 different days. The growth rates inferred from the experiment should be tabulated in the text or supplementary info, or shown on the figure itself, indicating the data points used to derive them.

Minor points and suggestions for the authors:

Introduction:
The sentence in the introduction about PHB may be a bit misleading. The 100x improvement (Dalia et al) the authors mention should be taken in the context of natural production, with appropriate conditions, being a further 10x higher than the level reached with the "100x" increase. The achievement of PHB content manipulation was definitely significant, not because of the absolute titers but because it showed the engineerability of the strain using the MuGENT method. This could be clarified in how the work is glossed here.

The authors could consider more explicitly explaining the difference between a GSMM and the previously published (Hoffart 2017, Long 2017) flux analysis models, either in terms of how the model is built and/or the usefulness of the resulting model. This could further help non-specialists understand the advantages of a GSMM and highlight the novelty of this manuscript.

Results & Discussion:
In the comparison of the GSMM to V. vulnificus and V. cholerae models, it would strengthen the point being made to give the number of predicted genes, and/or predicted metabolic genes based on GO classification, for each species. It could also be appropriate to compare the GSMM to a well-developed E. coli model like the one used further on in the text, iJO1366, since metabolism of Vibrio spp is overall less well understood than E coli's. This information could also be added to Fig 1cd.

"We used this value to constrain the substrate uptake rate in simulations of growth on minimal glucose medium." Would it be useful to briefly describe the sensitivity of the growth rate and biomass yield to the glucose uptake and acetate secretion rate constraints used? If the details of how modeling is done makes this impractical or meaningless it might be helpful to less experienced readers like me to spell out why that's the case. Similarly, glucose > G6P is not subject to FVA in Fig 2, which I assume is because it's constrained, but it's not obvious to a non-specialist why that constraint is required, other than that glucose uptake rate is the major determinant of growth rate.

Comparison to E. coli section: the idea that V. natriegens "holds no obvious intrinsic metabolic advantage" vs. E. coli in terms of growth rate would be better supported by comparing the 3.9 g/(g*h) glucose uptake rate to experimental data for E. coli glucose uptake. If this glucose uptake rate is atypical for E. coli it might be helpful to rewrite the discussion of the metabolic model result here a bit to take that into account.

In Figure 1b, 13C should use a superscript for the 13.

In Figure 2a, the y-axes need titles, and should be scaled so that the highest and lowest y bars are fully inside the frame rather than touching it (or an axis break included if the actual y-value is currently outside the plot). The x-axes could be clearer if the rxn and _c0 characters repeated in every label were omitted and mentioned in the caption instead.

In the Figure 2b caption, the authors may want to clarify whether the fluxes shown are the experimental data from Long et al. since "adapted from Long et al. 2017" is slightly ambiguous, and some arrow widths don't seem to match the dots in Fig 2a, for example rxn00154_c0 and rxn00148_c0 which are very different in 2a but similar in 2b. Are the fluxes shown actually the average values from the FVA results in 2a?

"A GSMM cannot capture this crabtree-like behaviour of V. natriegens, which secretes acetate instead of redirecting carbon towards the TCA." Crabtree should be capitalized.

"The model, attempting to optimise biomass formation, naturally prefers redirecting carbon towards the TCA over secreting acetate." This is a little confusing in light of the earlier statement that acetate secretion was fixed at an experimentally determined rate. Whether this constraint was imposed during FVA should be clarified.

"The model predicts similar growth rates (~1.6 h-1) on glucose, galactose, rhamnose, maltose, glycerol, fructose, sucrose, glucosamine and N-acetylglucosamine." How were the uptake rates for these sugars determined or set? Since the text earlier mentions fixing the glucose uptake rate to match experiments, the same issue should be addressed here too. The text does mention "variations in transporter-mediated substrate uptake rates" but the difference in how glucose vs. other sugars were tackled should be clarified.

"In addition, we experimentally assessed V. natriegens' ability to use 190 distinct carbon sources. For each carbon source represented in the model, in silico growth was determined..." This is a bit confusing as written. Mentioning the platform/approach used to do this in the text (even though that duplicates the Methods) would help make it clearer, and briefly describing / summarizing the experimental results, for example giving the total number of growth vs. no growth conditions, before discussing the in silico work would make the distinction between the methods and data sets clearer. It also seems important to introduce the method in the text because details of how it works are explicitly called out later in the paragraph ("Firstly, it is important to notice that the experimental data quantifies respiration and not growth").

Table 1 caption: the phrase "strongest homolog" should be changed to "closest" or "most similar" homolog.

Figure 4a might be clearer if the label "Anaplerotic carboxylations" were replaced with "PEP carboxylase" since the other labels in the figure refer to proteins/enzymes. A separate label could show that both the PEP carboxylase and oxaloacetate decarboxylase reactions are anaplerotic carboxylations.

Methods, Generation of V. natriegens KO strain:
The sentence "The following program was run: 98 C for 1 minute, then 11 cycles of : 9 C for 1 s, 75 C for 1 s, Ta - ramped up to at - 0.1 C/sec, hold for 30 s. 72 C for 10 minutes" is a little unclear -- what was the initial and final Ta? Was the 1s temperature 9 or 98 C?

During cross-commenting, Reviewer #2 made additional comments and suggestions on how to improve the study, which I include below:

"My main concern here is about the validation of the model, especially since many researchers could use it for engineering or synthetic biology purposes. This is the first model for this organism and the foundation should be solid for others to confidently build on it.

A few web platforms (RAST/Seed, BioCyc, etc.) use automated pipelines to generate metabolic reconstructions. In my experience, these reconstructions comprise a significantly higher number of genes/reactions than heavily curated models. The authors mention that their model is more extensive than published models for Vibrio cholerae and Vibrio vulnificus. The difference is striking with 400-600 additional reactions in the V. natrigens model. One explanation for this observation is that the automatic reconstruction algorithms are less stringent, which can introduce errors and lead to false conclusions. So, is the V. natriegens model a more exhaustive and accurate representation of the metabolism or is it including reactions that are not genuinely present or inaccurate compared to other Vibrio models? A comparison between the Vibrio models would be very useful but was not reported. It would also be important to clearly describe the biomass objective function (what the cell is trying to build in growth simulation). This is not discussed in the manuscript and could influence the results.

One way to validate the model is to compare gene essentiality predicted by the model to experimental data. This is achieved in the manuscript using CRISPRi data. However, the metrics are not clearly presented. What is the accuracy of the model (what is the percentage of essential genes determined experimentally that are predicted to be essential by the model)? I understand that many factors (presented by the authors) could complicate this analysis, and this is why I mentioned transposon mutagenesis in my evaluation. Data is already available in the article of Lee et al (2019). Although the mutagenesis is not at saturation, there is valuable information to extract for model validation, with clear metrics to present.

Another validation in this context would be to compare predicted fluxes in the model with gene expression data (transcriptomic and ribosome profiling data is available for V. natriegens and is not so difficult to generate if for any reason published data is not appropriate). The point here would be to see if the genes required for a reaction to occur are expressed in a coherent fashion.

If these validations are not presented, it is difficult in my view to be confident about the predictions of the model (would the community really trust and use this model if the overall accuracy was low?). I would also like to add that the Na+-transporting oxaloacetate decarboxylase (Na+-OAD) gene deletion was not complemented to see if the growth defect was rescued when the gene is re-introduced in the strain.

For these reasons, the work as submitted remains preliminary in my opinion and should be better supported prior to publication. I see value in fast publication, but I think we will agree that this should not be at the cost of validations or quality control. I also have doubts about the level of biological novelty to justify a publication in Molecular Systems Biology. "

We thank the editors and reviewers for time dedicated to our manuscript and the valuable comments and suggestions. To improve our manuscript, we have addressed each concern to the best of our capability by complementing our paper with novel *V. natriegens* experimental data, comparative analyses and revised simulations:

- Metabolite and reaction ID mapping to other platforms
- Quality control of our GSMM using MEMOTE
- Revised manual curation of all reactions in the modelSEED draft reconstruction
- Generation of novel proteomics data to further validate the model
- Extensive metabolic comparison to model organism *E. coli*
- Construction and experimentation with a complementation strain for the OAD KO.

Furthermore, in addition to providing what was asked by the reviewers in the previous iteration, we have added significant amounts of new content in the revised version of the manuscript:

- Differential expression analysis of proteomics data between 0 mM and 300 mM NaCl to gain insights in halophilic adaptations of *V. natriegens*
- Development and publication of a resource balance analysis (RBA) model
- Analysis of growth simulations with the RBA model

Together, we believe these new results enhance the scientific novelty and contribution of our work. We expect the model to be a valuable resource for the scientific community.

MAJOR CONCERNS:

--Memote is a useful tool for quality control and to ensure that the best practices already used in software development are adopted. The draft reconstruction was automatically built with the SEED server and manually curated (potentially flawed in quality, ensure compatibility with other model identifiers). The number of reactions that were gap-filled by SEED would be important to present here. It would also be important to clearly indicate if Memote was run on this metabolic model reconstruction. Also, metabolic model names usually include the number of genes and not the number of metabolites as presented here.

We thank the reviewer for this suggestion to use MEMOTE as an additional way to validate the quality of our model. We perfomed mapping of metabolites and reactions to ensure compatibility across platforms (lines 140-142: "A mapping was done of metabolite, reaction, and gene ids to identifiers across online biochemistry databases to ensure cross-database compatibility.").

We reported on a MEMOTE score of 90% which is about the same as the benchmark *E. coli* model iJO1366 (lines 142-146: "The quality of the GSMM was assessed using the MEMOTE software suite, which has been developed as a benchmarking and quality control tool for GSMMs that also assesses cross-compatibility with various databases (Lieven et al., 2020). The MEMOTE software reported a score of 90%, which is comparable to the 91% yielded by MEMOTE for the benchmark Escherichia coli model iJO1366").

We performed all gapfilling manually so have specified in the materials and methods section that SEED gapfilling was not used (lines 621-624: "We did not use the gapfilling option provided by SEED. Instead, missing reactions were manually added to the network based on knowledge retrieved from the RAST annotation, NCBI BLAST (Altschul *et al.*, 1990) and the KEGG (Kanehisa *et al.*, 2012) database").

We have also changed the name of the model to iLC858, to include the number of genes rather than the number of reactions.

--There is no detailed comparison to other Vibrio metabolic models. The authors highlight the greater number of metabolites in their reconstruction but do not show more substantial comparisons with related Vibrio models. It would be important to explain the main differences between these Vibrio models in addition to E. coli (the biomass composition could also be compared to iML1515 instead of iJO166). What about the number of shared reactions between these models? Was the ID mapping performed? Flux variability analysis? Shadow prices of the models?

We thank the reviewer for these suggestions. We have performed a stricter curation of our model by manually checking at all the reactions suggested by SEED that did not have any equivalents in the *Vibrio cholerae* model iAM-Vc960, which resulted in removal of many reactions and metabolites (now clarified in lines 131-134: "The biomass reaction suggested by the SEED server was kept in the model. All reactions that had been included by the SEED server of which no equivalents were found in the *V. cholerae* model iAM-Vc960 (Abdel-Haleem *et al.*, 2020) were manually checked and curated").

As a result, our model has shrunk to a similar size (982 cytoplasmatic reactions) as the *Vibrio cholerae* model (1,016 cytoplasmatic reactions).

We have now also performed ID mapping and included this as a supplementary file with our manuscript (entire paragraph lines 153-155: "We also performed ID mapping of reactions from iLC858 to iAM-Vc960 and have included this as Supplementary file 2.").

--Additional experimental validations would be needed to fully support the model. For example, Page 4, line 6: "Even under aerobic conditions, V. natriegens secretes part of its substrate as acetate, indicating a mixed respiratory-fermentative growth phenotype". This would be an interesting experimental validation to perform that is not excessively complicated. On the same idea, the fluxes observed in the model could be compared to the expression of the corresponding enzymes using transcriptomics or ribosome profiling datasets (see this dataset as an example: https://www.ncbi.nlm.nih.gov/geo/query/acc.cgi?acc=GSM2500126). This would provide another metric to evaluate the model compared to experimental data. Transposon mutagenesis, although not absolutely required, would also be an alternative or complement the CRISPRi data.

We thank the reviewer for these suggestions. We considered performing additional experiments to quantify acetate in aerobic condition, but these results were confirmed in the literature in a consistent and independent manner on minimal medium by Long et al., (0.27 g acetate / g glucose, 2017) and Hoffart et al. (0.41 g acetate / g glucose, 2017).
However, to improve the manuscript and model validation, we have generated and analysed proteomics data of a variety of samples in different salinities.
We identified in silico 390 GSMM reactions that can produce fluxes above a threshold of 10^{-6} mmol $g^{-1}.h^{-1}$, of which 300 reactions (76.9%) had associated gene whose corresponding enzymatic machinery was entirely found in at least one of the proteomics samples. This validation is more extensively covered in lines 333-339: "Flux sampling was used to determine reactions in the GSMM which are predicted to be able to produce fluxes of at least 10^{-6} mmol $g^{-1}.h^{-1}$, a threshold taken from Altea-Manzano *et al.* (2020). Across 100 FBA samples of *V. natriegens* aerobic growth, 390 such reactions were identified. Of these, 300 reactions (76.9%) had associated gene whose corresponding enzymatic machinery was entirely found in at least one of the proteomics samples. Moreover, 331 reactions (84.9%) had associated gene whose all enzymatic subunits except one were found in at least one of the proteomics samples.".

The quality of the predictions for gene essentiality should be delivered by calculating accuracy and precision. The Matthews correlation coefficient should also be considered as a metric for the quality of predictions. As currently presented, it is difficult to clearly obtain these metrics.

Thank you for the suggestion. We have now revised the gene essentiality section and limited our conclusions in the text since the only experimental dataset available (HTP pooled CRISPRi by Lee et al.) does not look at true knock-out and has been reported by the authors to lack exact reproducibility. Still, we have included the accuracy of the predictions (lines 312-313: "Of these, 83 (49%) were also found by the in silico analysis as 'essential genes'"). Future *V. natriegens* knock-out libraries will help us better validate our model.

--Page 5, line 28: "The model predicts similar growth rates (~1.6 h-1) on glucose, galactose, rhamnose, maltose, glycerol, fructose, sucrose, glucosamine and N-acetylglucosamine. This demonstrates that the enzymes required to utilize these carbon sources at similar efficiencies as glucose are encoded within the V. natriegens genome". This indicates that the genes catalyzing these reactions were found in the genome and that the reactions responsible for their degradation have a similar stoichiometry. The bounds on the model were not changed and the exchange flux (not mentioned) was probably maintained at 3.9 like for glucose. A kinetic model and catalytic constants would be required to make such a claim.

We thank the reviewer for pointing out the inaccuracy of this claim. We have now reformulated this to make the right claim, being that the enzymes required to utilise these carbon sources at similar stoichiometries as glucose are encoded within the V. natriegens genome (lines 232-234: "This demonstrates that the enzymes required to utilise these carbon sources at similar stoichiometries as glucose are encoded within the *V. natriegens* genome")

MINOR COMMENTS:

-I would suggest using page and/or line numbers in future manuscript submissions to facilitate the review process.

Thank you for the suggestion. We have now added line numbers to our manuscript.

-Page 7, line 1: "The vast majority (92.8%) of reactions in our model, not counting passive transmembrane transport reactions or metabolite sink reactions, are associated with at least one known coding sequence in the V. natriegens genome". Details about this should be presented earlier in the model reconstruction section:

Thank you for the suggestion. We have now presented the fraction of gene associated reactions in the model reconstruction section (lines 138-140: "The vast majority (90.1%) of reactions in our model, not counting passive transmembrane transport reactions or metabolite sink reactions, are associated with at least one known coding sequence in the *V. natriegens* genome.").

--Figure 1. Please cite directly on the figure the source for the data that was used to validate the model. As currently represented, this suggests that the data was generated exclusively for this paper.

This is a good point. We have now cited directly on the figure the sources of the validation data.

--Figure 2. a) Choosing a color other than blue would accentuate the contrast between the grey lines and the dots. Reaction names (human-readable) would be easier to understand instead of SEED identifiers. Units should be represented on the Y-axis. Coloring reactions by subsystem would facilitate the interpretation of this graph.
b) How do model predictions compare with experimental observations here? As currently represented, it is not possible to get this information.

These are good suggestions, thank you. A) We have now accentuated dots by increasing their size, labelled the reactions with human readable names, coloured the reactions by subsystem and noted the units on the Y-axis. B) We have now reported on a high Pearson's correlation coefficient between the model's optimal prediction and experimental fluxes (lines 197-198: "Comparing the fluxes predicted by the model's optimal solution and experimental fluxes yielded a high Pearson's correlation coefficient of 99.2%").

--Figure 3. b) and c) The quality of the predictions should be delivered by calculating accuracy and precision. The Matthews correlation coefficient should also be considered as a metric for the quality of predictions.

Thank you for the suggestion. We have now presented the accuracy as a metric for the quality of the carbon substrate predictions in the figure (lines 262-263: "The model predicted growth for 36 of 81 (44,4%) carbon sources on which *V. natriegens* can grow and predicted non-viability for 107 of 109 (98,2%) carbon sources on *which V. natriegens* cannot grow, leading to an overall predictive accuracy of 75,3%.").

Reviewer #3:

The authors construct the first genome-scale metabolic model for the fast-growing bacterium Vibrio natriegens using available genome sequence data. They validate the curated model by showing impressive agreement between the predicted growth values and metabolic fluxes and the corresponding experimental results from Hoffart et al and Long et al., and also make comparisons with gene essentiality data from Lee et al. and the authors' own carbon source utilization results, showing very reasonable levels of agreement in both cases, including plausible suggestions about sources of disagreement. They take initial steps to experimentally test an interesting functional prediction of their model, that an oxaloacetate decarboxylase (Na+-OAD pump) may play an important role in aerobic growth in high salinity, in a function supported by previous biochemical work. Overall, the authors provide a compelling description and demonstration of the value of GSMM building and how it could influence experimental engineering or evolution efforts. The methods used do not seem technically innovative but they are rigorously applied and the results represent a significant contribution to understanding and manipulating this emerging model organism. The paper should be of interest to those working in Vibrio biology, metabolic engineering in general (since it highlights and enables the usefulness of V natriegens as a new chassis), and metabolic biochemistry. I strongly recommend this paper for publication, subject to the following revisions.

Major points - straightforward but important to correct:
The authors say: "It is possible then that the ED pathway may be activated as an alternative to glycolysis depending on the carbon source, as is the case when E. coli is grown on gluconate (Eisenberg and Dobrogosz, 1967), or generally depending on V. natriegens' energy supply (Flamholz et al., 2013)"
Eagon and Wang, J Bacteriol 1961, give direct experimental evidence that ED enzymes are activated by growth on gluconate! So the speculation here seems to be correct, and the reference should be cited and discussed.

This excellent suggestion is very relevant to our paper. We thank the reviewer for pointing out what we overlooked in the literature. We have now referenced this work in our manuscript (lines 214-216: "This is consistent with early findings done by Eagon and Wang (Eagon and Wang (1962) who detected the enzymes of the ED pathway in *V. natriegens* when grown on gluconate").

Although I am not enough of a proteomics specialist to say for sure, the data underlying the claim that the Na+-OAD is differentially expressed in low and high salt conditions seems to need more support, qualification or explanation. The 5x differential expression figure seems to come from raw counts of peptides but the methods don't describe measures to normalize for total protein content of the samples or to place Na+-OAD levels in the context of overall protein expression. The differential expression claim is not unreasonable given the

knockout/growth data and the modeling results, which are persuasive, so I might suggest discussing it more tentatively in the text, or, if the proteomics methods used are indeed standard and reasonable in the context of the technique, this could be spelled out in the text and perhaps also the methods, to support the idea that differences in peptide count are a good proxy for differential expression.

We thank the reviewer for pointing out the lack of support of the OAD being differentially expressed. We agreed that this needed further investigation and have therefore repeated and expanded our proteomics data since the original submission of the manuscript. In this new dataset we still find OAD being expressed in aerobic conditions, but differential expression was not statistically significant between different salinities. We have adapted this in the text and attempted to give a clearer explanation of the proteomics (lines 451-454: "While we did not find this pump to be significantly differentially expressed between different salinities in the proteomics samples, it was highly expressed in all of them, reaching expression levels in the same order of magnitude as the Na^+-translocating NADH:ubiquinone oxidoreductase."). This suggest that the effects found in growth rate when OAD was knocked out in high salinity would be regulated at the enzymatic level. We have now discussed this in the text ("Alternatively, a further explanation of why the functionality of the Na^+-OAD is limited to high salinity environments could be that a low salinity environment may simply not be able to provide the strong sodium gradient that has been described to be required to activate the carboxylation function of the pump (Dimroth and Hilpert, 1984).") We thank the reviewer for helping us improve the scientific accuracy of our findings.

The Figure 4b caption and figure don't make clear that the right-hand panel is under high salinity and the left-hand panel low salinity. The figure panels should have separate captions or be labelled clearly on the figure itself, and the caption should give the salinity used. The methods seem to imply that this would be given in the figure/caption and it's missing; it should be in both places. The meaning of the -1, -2 and -3 conditions should be spelled out in the caption, not just in the methods (assuming these are the triplicate wells from a single experiment). Ideally, replicates from a single experiment would be averaged and the entire experiment repeated on {greater than or equal to}3 different days. The growth rates inferred from the experiment should be tabulated in the text or supplementary info, or shown on the figure itself, indicating the data points used to derive them.

We thank the reviewer for his feedback. We had indeed forgotten to specify salinity levels on the plots. We have now regenerated those growth curves in the lab and found the same conclusions as previously. We have updated the figure to include correct labelling of salinity as well as being the mean of the biological replicates and provided more information on this experiment as Supplementary file 9.

Minor points and suggestions for the authors:

Introduction:
The sentence in the introduction about PHB may be a bit misleading. The 100x improvement (Dalia et al) the authors mention should be taken in the context of natural production, with appropriate conditions, being a further 10x higher than the level reached with the "100x" increase. The achievement of PHB content manipulation was definitely significant, not because of the absolute titers but because it showed the engineerability of the strain using the MuGENT method. This could be clarified in how the work is glossed here.

We understand how this sentence could be misleading. We have now adapted this sentence to be more representative of the meaningfulness of the MuGENT method (lines 79-83: "Furthermore, a clever new genetic engineering method called MuGENT was designed to facilitate metabolic engineering for bioproduction requiring multiple genomic edits. This technique was subsequently leveraged to increase V. natriegens' natural ability to accumulate the bioplastic precursor poly-☐-hydroxybutyrate (PHB) (Chien et al., 2007; Dalia et al., 2017)").

The authors could consider more explicitly explaining the difference between a GSMM and the previously published (Hoffart 2017, Long 2017) flux analysis models, either in terms of how the model is built and/or the usefulness of the resulting model. This could further help non-specialists understand the advantages of a GSMM and highlight the novelty of this manuscript.

We thank the reviewer for this suggestion. We have now more explicitly explained how GSMM's scope goes beyond the model presented by Long (2017) in the introduction (lines 106-111: "A minimal flux analysis model of the central metabolism, containing 42 reactions, had already been presented by Long *et al.* (2017). The scope of a GSMM goes far beyond the central metabolism, as they aim to represent as many of the enzymes encoded on an organism's genome as possible. This makes GSMMs useful tools for analysis of non-central pathways, substrate viability prediction and gene essentiality analysis, which can not be done with metabolic models limited to central metabolism.").

Results & Discussion:
In the comparison of the GSMM to V. vulnificus and V. cholerae models, it would strengthen the point being made to give the number of predicted genes, and/or predicted metabolic genes based on GO classification, for

each species. It could also be appropriate to compare the GSMM to a well-developed E. coli model like the one used further on in the text, iJO1366, since metabolism of Vibrio spp is overall less well understood than E coli's. This information could also be added to Fig 1cd.

Thank you for the suggestions. We have now added a comparison to the size of *E. coli* model iJO1366 in the text as well as in figure 1 (lines 155-157: "Compared to the highly curated *E. coli* model iJO1366, our model iLC858 is still quite limited in size. This is expected, as only 858 *V. natriegens* genes are accounted for in our model, compared to the 1366 genes accounted for in iJO1366.").

"We used this value to constrain the substrate uptake rate in simulations of growth on minimal glucose medium." Would it be useful to briefly describe the sensitivity of the growth rate and biomass yield to the glucose uptake and acetate secretion rate constraints used? If the details of how modeling is done makes this impractical or meaningless it might be helpful to less experienced readers like me to spell out why that's the case. Similarly, glucose > G6P is not subject to FVA in Fig 2, which I assume is because it's constrained, but it's not obvious to a non-specialist why that constraint is required, other than that glucose uptake rate is the major determinant of growth rate.

Thank you for the feedback. We have now clarified in this section why those constraints are necessary (lines 162-165: "When solving a GSMM using Flux Balance Analysis (FBA), the model will maximally utilise any provided sources of carbon in order to generate biomass. Hence, constraints on substrate uptake rate and secretion of metabolites must be imposed to generate realistic simulations."). The reason that it appears that glucose -> G6P is not subjected to FVA is that this entry point of carbon has no flexible range within which it may vary without affecting the optimal solution. Any glucose uptake lower than the imposed constraint would consequentially lower the amount of biomass that can be generated. We have now updated this section (lines 194-196: "For this simulation, the same glucose uptake rate and acetate secretion rate as in the aerobic growth simulations were used. The predicted fluxes were represented as percentages of the glucose uptake rate, in order to enable comparison to Long's data.").

Comparison to E. coli section: the idea that V. natriegens "holds no obvious intrinsic metabolic advantage" vs. E. coli in terms of growth rate would be better supported by comparing the 3.9 g/(g*h) glucose uptake rate to experimental data for E. coli glucose uptake. If this glucose uptake rate is atypical for E. coli it might be helpful to rewrite the discussion of the metabolic model result here a bit to take that into account.

We thank the reviewer for pointing this out. We have now removed this statement due to lack of clarity. Additionally, we have expanded this section to contain a more extensive comparison between the *V. natriegens* and *E. coli* models. (entire section "A metabolic comparison with model organism *E. coli* ": lines 341-388)

In Figure 1b, 13C should use a superscript for the 13.

Thanks for pointing this out. We have now adapted this in figure 1.

In Figure 2a, the y-axes need titles, and should be scaled so that the highest and lowest y bars are fully inside the frame rather than touching it (or an axis break included if the actual y-value is currently outside the plot). The x-axes could be clearer if the rxn and _c0 characters repeated in every label were omitted and mentioned in the caption instead.

Thanks for the suggestion. We have now improved the figure with the reviewer's suggestions. The curations we have made to improve the model have now elegantly resulted in FVA ranges that fall within the 0-200 range of the plot.

In the Figure 2b caption, the authors may want to clarify whether the fluxes shown are the experimental data from Long et al. since "adapted from Long et al. 2017" is slightly ambiguous, and some arrow widths don't seem to match the dots in Fig 2a, for example rxn00154_c0 and rxn00148_c0 which are very different in 2a but similar in 2b. Are the fluxes shown actually the average values from the FVA results in 2a?

Thank you for pointing out this ambiguity. We have made clear in the caption that the only adaptation made to Long's figure is a colourisation of the arrows. The fluxes shown are the actual fluxes published by Long et al. 2017. The reason the arrow of rxn00148 (now CDC19) did not match the arrow provided by Long is that the conversion of PEP -> pyruvate happens with two reactions, namely the import of glucose through PEP-mediated transport as well as the normal pyruvate kinase reaction. In our previous FVA we had only accounted for the pyruvate kinase, while Long's arrow represents both reactions. We have now also included both reactions for our FVA analysis and clarified this better in the figure.

"A GSMM cannot capture this crabtree-like behaviour of V. natriegens, which secretes acetate instead of redirecting carbon towards the TCA." Crabtree should be capitalized.

We have now capitalised Crabtree wherever it occurred in the text.

"The model, attempting to optimise biomass formation, naturally prefers redirecting carbon towards the TCA over secreting acetate." This is a little confusing in light of the earlier statement that acetate secretion was fixed at an experimentally determined rate. Whether this constraint was imposed during FVA should be clarified.

Thank you for pointing this out. We had indeed used this acetate secretion constraint during FVA and have now made this clear in the text (lines 194-195: "For this simulation, the same glucose uptake rate and acetate secretion rate as in the aerobic growth simulations were used.").

"The model predicts similar growth rates (~1.6 h-1) on glucose, galactose, rhamnose, maltose, glycerol, fructose, sucrose, glucosamine and N-acetylglucosamine." How were the uptake rates for these sugars determined or set? Since the text earlier mentions fixing the glucose uptake rate to match experiments, the same issue should be addressed here too. The text does mention "variations in transporter-mediated substrate uptake rates" but the difference in how glucose vs. other sugars were tackled should be clarified.

Substrate uptake rates are not determined for these substrates so we constrained the model to take up carbon at an equivalent rate as glucose in terms of carbon units. We acknowledge our lack of clarity here and have elaborated on this in the manuscript (lines 229-232: "When a carbon-equivalent uptake rate to glucose is used as a constraint, the model predicts similar growth rates (~1.6 h^{-1}) on glucose, galactose, rhamnose, maltose, glycerol, fructose, sucrose, glucosamine and N-acetylglucosamine").

"In addition, we experimentally assessed V. natriegens' ability to use 190 distinct carbon sources. For each carbon source represented in the model, in silico growth was determined..." This is a bit confusing as written. Mentioning the platform/approach used to do this in the text (even though that duplicates the Methods) would help make it clearer, and briefly describing / summarizing the experimental results, for example giving the total number of growth vs. no growth conditions, before discussing the in silico work would make the distinction between the methods and data sets clearer. It also seems important to introduce the method in the text because details of how it works are explicitly called out later in the paragraph ("Firstly, it is important to notice that the experimental data quantifies respiration and not growth").

We understand how our writing here can cause confusion and have clarified this bit using the reviewer's suggestions (lines 255-258: "In addition, we experimentally assessed V. natriegens' ability to use 190 distinct carbon sources using BioLog Phenotype MicroArrays, a high-throughput assay which measures respiration as a proxy for growth. 81 of 190 were found to be viable carbon substrates for V. natriegens*").*

Table 1 caption: the phrase "strongest homolog" should be changed to "closest" or "most similar" homolog.

We thank the reviewer for this suggestion and have made this adaptation to the manuscript.

Figure 4a might be clearer if the label "Anaplerotic carboxylations" were replaced with "PEP carboxylase" since the other labels in the figure refer to proteins/enzymes. A separate label could show that both the PEP carboxylase and oxaloacetate decarboxylase reactions are anaplerotic carboxylations.

Thanks for the suggestion. We have adapted the figure to be more clearly distinct between the PEP carboxylase and the oxaloacetate decarboxylase.

Methods, Generation of V. natriegens KO strain:
The sentence "The following program was run: 98 C for 1 minute, then 11 cycles of : 9 C for 1 s, 75 C for 1 s, Ta - ramped up to at - 0.1 C/sec, hold for 30 s. 72 C for 10 minutes" is a little unclear -- what was the initial and final Ta? Was the 1s temperature 9 or 98 C?

Thanks for pointing out this ambiguity. We have now made this clearer in the text (lines 687-689: "The following program was run: 98 C for 1 minute, then 11 cycles of : 98 C for 1 s, 75 C for 1 s, Ta - ramped down to 72 C at - 0.1 C/s from 75 C , hold for 30 s. 72 C for 10 minutes").

During cross-commenting, Reviewer #2 made additional comments and suggestions on how to improve the study, which I include below:

"My main concern here is about the validation of the model, especially since many researchers could use it for engineering or synthetic biology purposes. This is the first model for this organism and the foundation should be solid for others to confidently build on it.

A few web platforms (RAST/Seed, BioCyc, etc.) use automated pipelines to generate metabolic reconstructions. In my experience, these reconstructions comprise a significantly higher number of genes/reactions than heavily curated models. The authors mention that their model is more extensive than published models for Vibrio cholerae and Vibrio vulnificus. The difference is striking with 400-600 additional reactions in the V. natrigens model. One explanation for this observation is that the automatic reconstruction algorithms are less stringent, which can introduce errors and lead to false conclusions. So, is the V. natriegens model a more exhaustive and accurate representation of the metabolism or is it including reactions that are not genuinely present or inaccurate compared to other Vibrio models? A comparison between the Vibrio models would be very useful but was not reported. It would also be important to clearly describe the biomass objective function (what the cell is trying to build in growth simulation). This is not discussed in the manuscript and could influence the results.

Thanks for the suggestion. We have improved the model by manually going through all the reactions that were predicted by SEED but did not have equivalents in the *V. cholerae* model. This has drastically reduced our model to be similar in size to the *V. cholerae* one. We have also performed reaction mapping to the *V. cholerae* model and included it as supplementary figure. By lack off better alternative – and considering that the *V. cholerae* biomass reaction was based on *E. coli* – we have decided to keep the biomass formulation generated by SEED.

One way to validate the model is to compare gene essentiality predicted by the model to experimental data. This is achieved in the manuscript using CRISPRi data. However, the metrics are not clearly presented. What is the accuracy of the model (what is the percentage of essential genes determined experimentally that are predicted to be essential by the model)? I understand that many factors (presented by the authors) could complicate this analysis, and this is why I mentioned transposon mutagenesis in my evaluation. Data is already available in the article of Lee et al (2019). Although the mutagenesis is not at saturation, there is valuable information to extract for model validation, with clear metrics to present.

Thanks for the suggestions. We have now reported the percentage of experimental essential genes (included in the model) that were also detected by the model to be essential. We have decided to not use the transposon mutagenesis data as the original author, Henry Lee, deemed it unsuitable as a source for gene essentiality in *V. natriegens.* (From Lee et al. 2019: We observed low saturation of transposon mutagenesis in V. natriegens, with only 47.7% of genes containing one insertion and 23.4% containing ≥2 insertions (Supplementary Table 8). Further analysis revealed that a large percentage of the transposon library sequencing reads mapped to the transposon backbone. This is indicative of genomic integration of the suicide transposon vector since no plasmids could be extracted from V. natriegens transconjugants, and direct electroporation of the transposon plasmid alone into V. natriegens did not produce any detectable transformants.
")

Another validation in this context would be to compare predicted fluxes in the model with gene expression data (transcriptomic and ribosome profiling data is available for V. natriegens and is not so difficult to generate if for any reason published data is not appropriate). The point here would be to see if the genes required for a reaction to occur are expressed in a coherent fashion.

Thanks for this suggestion. We have now added a comparison of predicted fluxes with a new and comprehensive proteomic dataset that we have generated for this study and found a good consistency between prediction and experiment. We have also reported on this (entire section lines 332-338: "Flux sampling was used to determine reactions in the GSMM which are predicted to be able to produce fluxes of at least 10^{-6} mmol $g^{-1}.h^{-1}$, a threshold taken from Altea-Manzano *et al.* (2020). Across 100 FBA samples of *V. natriegens* aerobic growth, 390 such reactions were identified. Of these, 300 reactions (76.9%) had associated gene whose corresponding enzymatic machinery was entirely found in at least one of the proteomics samples. Moreover, 331 reactions (84.9%) had associated gene whose all enzymatic subunits except one were found in at least one of the proteomics samples.").

If these validations are not presented, it is difficult in my view to be confident about the predictions of the model (would the community really trust and use this model if the overall accuracy was low?). I would also like to add that the Na+-transporting oxaloacetate decarboxylase (Na+-OAD) gene deletion was not complemented to see if the growth defect was rescued when the gene is re-introduced in the strain.

This was also a good suggestion, thanks. We have generated a complementation strain now and seen that the growth defect was indeed rescued. We have also reported on this in the manuscript (lines 442-446: “We therefore created a knockout (KO) strain for this gene, and a complementation strain harboring the re-inserted OAD gene. We generated growth curves for all three strains at 0 mM and 300 mM. We found that only under high salinity conditions, the OAD knock-out grew more slowly and to a lower OD than the WT and OAD complementation strain (Figure 5, Supplementary file 9).”).

For these reasons, the work as submitted remains preliminary in my opinion and should be better supported prior to publication. I see value in fast publication, but I think we will agree that this should not be at the cost of validations or quality control. I also have doubts about the level of biological novelty to justify a publication in Molecular Systems Biology. "

We agree that the quality of the manuscript should prevail and that is why we have now improved this article with over a year of experimental and computational work. We believe that the new validations, expansions of the model (RBA) and omics dataset have made the manuscript significantly better. In addition, during this year, we have presented our results in conferences and the *V. natriegens* community is excited about the release of the model that will be useful for those working on metabolic engineering and studying the physiology and metabolism of this interesting microorganism.

1st Revision - Editorial Decision **27th Sep 2022**

Thank you for sending us your revised manuscript. Since Reviewer #2 from the previous round of review was not available this time, we were obliged to seek advice from one new expert who could evaluate the modelling part. We have now heard back from the two reviewers who agreed to review your study. You will see from the comments below that while Reviewer #3 (who had reviewed the manuscript before) remains positive about the study, Reviewer #4 (who reviewed the manuscript for the first time) raises significant concerns regarding the model completeness and the limited novel mechanistic and biological insights derived from the model.

Under these circumstances and given that the concerns raised are substantial and are unlikely to be addressable within the scope of a major revision, we see no other choice than to return the manuscript with the message that we cannot offer to publish it.

While we cannot pursue this manuscript further, we encourage you to transfer your study to our not-for-profit open-access sister journal, Life Science Alliance (LSA). We shared your manuscript and the accompanying reviews with LSA Executive Editor, Eric Sawey, who is interested in these findings, and would like to publish this manuscript at LSA pending the following revisions:

- Address Reviewer 3's comments.
- Address Reviewer 4's comments by adding to the manuscript as you wish, perhaps acknowledging some of these outlined limitations in the manuscript.

Reviewer #3:

On the whole, the authors have done a marvelous job addressing my comments on the initial submission as well as those of the other reviewer and they have substantially improved the manuscript. The added elements of the manuscript improve both its value to the community and its accessibility to non-specialists in metabolic modeling. I strongly recommend publication, pending (see comments below) (1) correction of omitted parts of Fig 3, (2) a modest rephrasing of the strength of conclusions about the role of OAD and the language of "complementation", and (3) any final revisions the authors wish to make to address reviewer comments.

Major points:
---

(1) Fig 3d and Fig 3e appear to be missing from the revised manuscript file. The text discussion is clear enough without them, but of course they should be included. Given the quality of the other figures I expect these will be suitable.

(2) I'm not compelled by the approach to "complementing" the OAD knockout. I think about complementation as a way to rule out polar effects or other changes created at the knockout locus that could contribute to the knockout phenotype by showing that expression of the gene elsewhere, in ADDITION to the knockout, is sufficient to restore the wild-type phenotype. Restoration of the wild-type allele at the OAD locus, coupled with antibiotic cassette replacement of dns, does control for some aspects of the knockout, such as presence of a resistance cassette in the genome and perhaps undesired genetic changes at other positions, but I would call it a reversion rather than a complementation. I recommend changing the language used to describe this experiment accordingly, labeling the strain "reverted" rather than "complemented" for clarity. A more compelling experiment would be to insert OAD at an unrelated locus such as dns, or express it from a plasmid, in the genetic context of the OAD knockout. However, I don't consider the OAD knockout to be an especially central part of the value of this paper, and the knockout phenotype is in line with the GSMM prediction whether complementation is shown or not. It would be a mistake to delay publication for the sake of a more compelling complementation data set. Instead I recommend more qualified, more cautious statements about the evidence for the pump's contribution to the V. natriegens growth rate. The authors' data are consistent with their argument but are also consistent with a growth defect due to polar effects on the downstream genes.

Minor points and suggestions:

In a number of places the authors are not fully consistent in the use of . or , as a decimal place marker.

293 "growth rate reduced below 90%" This should be clarified: do the authors mean that the growth became <= 90% of its maximal value, or that it was reduced by >= 90% (becoming <= 10% of maximal)? Presumably it's the latter, but I can't tell!

301-315 The comparison with Lee et al is helpful, and the 49% agreement rate seems quite reasonable to me given the issues the authors note that would tend to generate false negatives for the GSMM essentiality prediction. The authors might suggest that manual examination of the sets of non-overlapping essential genes could provide insight into enzyme roles or specificities in V. natriegens (but I consider this suitable for a follow-up study, not needed to complete this manuscript). (I also note that the authors point toward this at 571-575, that may be sufficient.)

322 The authors might note that the commonly used V. natriegens cultivation medium "LB3" contains 530 mM NaCl.

324-325 The method of normalization isn't fully clear to me from the methods. What are spectrum counts normalized to?

355 Should read "transcripts encoding both enzymes"

403-407 Seems like the second sentence should refer to V. natriegens' Na+-OAD *singular*, not plural, since there is only one!

440-442 The wording here is slightly confusing -- I would add, "while at lower salinity, there was no difference in growth rate."

Table 1 I think the comparisons would be clearer if a gene identifier were used in each row in addition to the base positions of the BLAST hit.

531 The authors might also comment on the RBA uptake rate result of 2.67 g gDW-1 h-1 compared to the experimental / FBA value of 3.9.

586 I recommend the authors add "to our knowledge" to the statement that this is the first demonstration of the Na+-OAD pump's functionality. Given that complementation, in my view, is not convincingly shown here, I would also be more comfortable with a qualified, more cautious statement about the evidence for the pump's contribution to the growth rate. The authors' data are consistent with their argument but are also consistent with a growth defect due to polar effects on the downstream genes.

652 Restating the criteria from the text about viability determination would be helpful.

690-711 The methods would benefit from a report of the efficiency of the knockout (approximate number of resistant CFU compared to total CFU) and the results of colony screening - if multiple colonies were checked by PCR, were they all verified knockouts? What was the approximate efficiency of cotransformation, i.e. the fraction of AmpR colonies that were CmpS? In my view, the MuGENT protocol is not so well established in V. natriegens that it can't benefit from detailed reports on its effectiveness across multiple research groups.

717 should read "inoculated and grown overnight".
720 I would personally write "diluted" rather than "re-inoculated".

734-735 The conversion factor and wavelength used to convert OD to cell count should be given.

Fig 1 is a beautiful illustration of the workflow and results it presents.

Fig 2 is very clear.

Fig 3a The caption here could also mention that the sugar uptake rates were assumed to be the same as for glucose to make these model predictions -- this would help explain some of the discrepancies if the reader has skipped the text (which explains the results very clearly). Note that 3d and 3e referenced in the text are missing.

Fig 3c Should be 75.3% not 75,3%. The caption or the headers for the table could use a bit more information on the method for readers who have skipped the text, and especially there should be some clarification of what is meant by the different row titles in terms of growth prediction. It could be useful to present this data as a Venn diagram rather than a table.

Fig 4 The title and captions could use a little more description in my view.

Fig 5b could be made larger relative to 5a. Otherwise this looks clear and effective.

Reviewer #4:

I have reviewed the revised manuscript, the previous round of reviewer comments, and the point-by-point response of the authors. In this work, the authors present a metabolic network reconstruction for V. natriegens, an organism of considerable metabolic engineering interest due to its fast growth rate and possibility of natural competence. Thus, a metabolic model for the organism is of substantial interest. The authors manually curate a draft reconstruction, assess the accuracy of the model in predicting gene essentiality and growth on various substrate, production potential, and present an analysis of salt adaptation and resource allocation in the organism.

As the primary interest in the organism is metabolic engineering, understanding the production potential and high growth rate of the organism are of primary interest. In that respect, the authors are hitting all the right marks. I have no doubt that the basic metabolic functions of the model are reasonable (evidenced by central metabolic fluxes and comparison of active fluxes to observed proteomics data). However, I find that key outcomes of the work, both in model completeness and knowledge derived from the model analysis, are less than satisfying. While this is a valuable first step in understanding V. natriegens, I'm not sure it will catch a lot of interest in its current form without answering more fundamental questions about the potential of the organism for metabolic engineering, specifically around the origin of its high growth rate and possibility for production outside of well-studied pathways.

Major comments

- The model has only 858 metabolic genes (compared to 1515 in the latest E. coli reconstruction) and predicts only 44% of growth on substrates, despite a similar overall genome size to E. coli, indicating that it is missing a substantial amount of metabolic content still. This is also relevant for the assessment of production potential - the authors look at amino acids and biomass precursors, which presumably have well-annotated pathways that don't change much in yield across organisms, but production potential of a wider array of substrates would be more interesting to assess the genetically-encoded metabolic capabilities of this organism. However if the current annotation is still limited, this analysis seems to be unlikely to be successful.
- The model performance stats are somewhat incomplete in their presentation (e.g. around line 307) and should include a full confusion matrix for essentiality and a more detailed breakdown of substrate-specific growth predictions and data. It currently seems like the authors are presenting the upside of their model performance, when a more unbiased presentation of performance would be preferred.
- The comparison of model-predicted growth rates to data in Figure 3a seems flawed if I understand the methodology correctly. In Line 225 the authors: "When a carbon-equivalent uptake rate to glucose is used as a constraint" - What is the basis for this assumption? It is best practice to measure the uptake rate of each substrate to make a quantitative growth rate prediction with FBA, or just make a binary growth/no-growth call.
- The resource balance analysis (RBA) seems to be a very promising tool to understand the high growth rate of Vibrio compared to E. coli and other platform organisms, but I find the section to be lacking any real conclusions. The model was somewhat arbitrarily parameterized to enable the observed growth rates, but there does not seem to be any in vitro evidence that the default catalytic rate should be 619200 h-1, for example, so the exercise seems to become somewhat arbitrary. If there was suggestion of an actual mechanism for increased growth rate, ideally with some kind of validation, this section would be much more interesting, and indeed answering a critical question for this organism as it is being considered for metabolic engineering applications.

Minor comments
- The list of model manual curations in Supplementary File 1 has no justification, and with lack of human readable reaction identifiers, it is currently incomprehensible
- The authors claim a MEMOTE overall score of 90% - is there any breakdown of performance stats that would make this value more interpretable to the reader? What does this mean?
- The authors repeatedly compare to iJO1366 but iML1515 is the latest version of the E. coli reconstruction, out for several years now

Authors appealed the decision.

Thank you for your message asking us to reconsider our decision regarding your manuscript MSB-2021-10523. I have now had a chance to read your preliminary point-by-point response and have also discussed them with the other members of the team. Based on the outline you provided, we think the proposed revisions sound reasonable.

As you may already know, our editorial policy allows, in principle, a single round of major revision. However, in this case, we think we can give you a chance to address them in an exceptional second round of major revision, provided that the issues raised by the reviewers can be convincingly addressed. Please note that the revised manuscript will be reviewed once again; it is therefore essential to provide responses to the reviewers' comments that are as complete as possible. Related to Referee #4's point #2, we would encourage you to include a full confusion matrix as a supplementary figure and discuss the potential limitation of the data, as mentioned in the revision plan.

On a more editorial level, please do the following:

We thank the editor for offering the opportunity to resubmit our manuscript and the reviewers for the time they dedicated to revise our work and suggest valuable ways to improve it. We have now addressed each of the concerns raised by reviewers #3 and #4. We have followed every piece of advice provided by reviewer #3. For #4, we have tried to address his concerns to the best of our capacity, which has significantly help improve the manuscript. In particular we have improved the curation of the biology substrate assays, provided gene essentiality confusion matrix, performed additional experiments to calculate biomass yields on various carbon sources and comparison with model prediction, and included an overview of all findings of this work possibly pertaining to V. natriegens' fast growth in the conclusion.

Reviewer #3:

On the whole, the authors have done a marvelous job addressing my comments on the initial submission as well as those of the other reviewer and they have substantially improved the manuscript. The added elements of the manuscript improve both its value to the community and its accessibility to non-specialists in metabolic modeling. I strongly recommend publication, pending (see comments below) (1) correction of omitted parts of Fig 3, (2) a modest rephrasing of the strength of conclusions about the role of OAD and the language of "complementation", and (3) any final revisions the authors wish to make to address reviewer comments.

Major points:
---
(1) Fig 3d and Fig 3e appear to be missing from the revised manuscript file. The text discussion is clear enough without them, but of course they should be included. Given the quality of the other figures I expect these will be suitable.

Thanks for pointing this out. We have now re-introduced the previous figure 3D and referred to it in the text. We replaced the previous figure 3E with a confusion matrix that has been provided as supplementary material in the context of requests made by other reviewers.

(2) I'm not compelled by the approach to "complementing" the OAD knockout. I think about complementation as a way to rule out polar effects or other changes created at the knockout locus that could contribute to the knockout phenotype by showing that expression of the gene elsewhere, in ADDITION to the knockout, is sufficient to restore the wild-type phenotype. Restoration of the wild-type allele at the OAD locus, coupled with antibiotic cassette replacement of dns, does control for some aspects of the knockout, such as presence of a resistance cassette in the genome and perhaps undesired genetic changes at other positions, but I would call it a reversion rather than a complementation. I recommend changing the language used to describe this experiment accordingly, labeling the strain "reverted" rather than "complemented" for clarity. A more compelling experiment would be to insert OAD at an unrelated locus such as dns, or express it from a plasmid, in the genetic context of the OAD knockout. However, I don't consider the OAD knockout to be an especially central part of the value of this paper, and the knockout phenotype is in line with the GSMM prediction whether complementation is shown or not. It would be a mistake to delay publication for the sake of a more compelling complementation data set. Instead I recommend more qualified, more cautious statements about the evidence for the pump's contribution to the V. natriegens growth rate. The authors' data are consistent with their argument but are also consistent with a growth defect due to polar effects on the downstream genes.

Thanks for your explanation. We have now changed the way we refer to the complementation strain into the reversion strain. We have done this both in the main text as well as in the supplementary file 11, going with this section. (lines 534-537: “We therefore created a knockout (KO) strain for this gene, and a reverted strain harbouring the re-inserted OAD gene. We found that only under high salinity conditions, the OAD knock-out grew slightly slower and to a lower OD than the WT and OAD reversion strain, while at lower salinity, we observed no difference in growth (Supplementary file 10)”)

Minor points and suggestions:

In a number of places the authors are not fully consistent in the use of . or , as a decimal place marker.

Thanks for pointing this out. We have updated the manuscript to be more consistent with decimal notation.

293 "growth rate reduced below 90%" This should be clarified: do the authors mean that the growth became <= 90% of its maximal value, or that it was reduced by >= 90% (becoming <= 10% of maximal)? Presumably it's the latter, but I can't tell!

Thanks for pointing out this ambiguity. We have now clarified this in the text (lines 364-366: "Knocking out 182 of 858 genes *in silico* resulted in an unsolvable FBA or growth rate reduced below 90% of the wild type growth rate, which we classified as 'essential'.").

301-315 The comparison with Lee et al is helpful, and the 49% agreement rate seems quite reasonable to me given the issues the authors note that would tend to generate false negatives for the GSMM essentiality prediction. The authors might suggest that manual examination of the sets of non-overlapping essential genes could provide insight into enzyme roles or specificities in V. natriegens (but I consider this suitable for a follow-up study, not needed to complete this manuscript). (I also note that the authors point toward this at 571-575, that may be sufficient.)

We have now added this suggestion to this section of the manuscript. (lines 390-392: "In future work, manual examination of the sets of non-overlapping essential genes could provide insight into enzymes roles or specificities in *V. natriegens."*).

322 The authors might note that the commonly used V. natriegens cultivation medium "LB3" contains 530 mM NaCl.

We have now added this information to this paragraph (lines 399-400: "For comparison, the most commonly used *V. natriegens* cultivation medium LB3 contains 530 mM NaCl")

324-325 The method of normalization isn't fully clear to me from the methods. What are spectrum counts normalized to?

We have now clarified the method of normalisation. (lines 1008-1010: "Spectrum counts were normalised using the standard Scaffold normalisation procedure which normalises the counts for each detected protein in a sample with respect to the total protein counts per analysed sample to allow cross-sample comparison").

355 Should read "transcripts encoding both enzymes"

The suggestions made by the reviewer is indeed more accurate so we have adapted this in the manuscript (lines 440-443: "Transcripts encoding both enzymes however were found with high ribosomal density in a ribosomal profiling dataset generated by Lalanne *et al.* (2018), where *V. natriegens* was grown in complete MOPS medium.")

403-407 Seems like the second sentence should refer to V. natriegens' Na+-OAD *singular*, not plural, since there is only one!

That is correct, thank you. We have now adapted this in the manuscript (lines 564-565: "Taken together, our *in silico* and experimental findings reveal a so far unknown, yet potentially relevant, role of the Na^+-OAD in *V. natriegens* metabolism.")

440-442 The wording here is slightly confusing -- I would add, "while at lower salinity, there was no difference in growth rate."

We have now incorporated the reviewer's suggestion to enhance clarity. (lines 536-538: "We found that only under high salinity conditions, the OAD knock-out grew slightly slower and to a lower OD than the WT and OAD reversion strain, while at lower salinity, we observed no difference in growth (Supplementary file 10).")

Table 1 I think the comparisons would be clearer if a gene identifier were used in each row in addition to the base positions of the BLAST hit.

We have now included ATCC 14048 gene identifiers to the blast hit coordinates in Table 1.

531 The authors might also comment on the RBA uptake rate result of 2.67 g gDW-1 h-1 compared to the experimental / FBA value of 3.9.

We have now commented on this substrate uptake results (line 634-637: "The predicted substrate uptake rate 2.67 $g.gDW^{-1}.h^{-1}$ is lower than the experimentally determined value but also corresponds to a lower growth rate, which might be explained by the more stringent constraints imposed by the RBA model. ").

586 I recommend the authors add "to our knowledge" to the statement that this is the first demonstration of the Na+-OAD pump's functionality. Given that complementation, in my view, is not convincingly shown here, I would also be more comfortable with a qualified, more cautious statement

about the evidence for the pump's contribution to the growth rate. The authors' data are consistent with their argument but are also consistent with a growth defect due to polar effects on the downstream genes.

We have followed the reviewer's recommendation and nuanced this in the text (lines 739-740: "To our knowledge, this is the first demonstration of the functionality of this enzyme during aerobic growth in bacteria").

652 Restating the criteria from the text about viability determination would be helpful.

We have followed the reviewer's recommendation and repeated the definition of viability in this paragraph. (lines 858-860: "The outcomes of both the *in silico* and experimental growth assays were classified as 'viable' or 'not viable', with viable being the models ability to produce biomass solely from the substrate in question as carbon source.").

690-711 The methods would benefit from a report of the efficiency of the knockout (approximate number of resistant CFU compared to total CFU) and the results of colony screening - if multiple colonies were checked by PCR, were they all verified knockouts? What was the approximate efficiency of cotransformation, i.e. the fraction of AmpR colonies that were CmpS? In my view, the MuGENT protocol is not so well established in V. natriegens that it can't benefit from detailed reports on its effectiveness across multiple research groups.

We agree that it is of interest to further systematically characterize the MuGENT protocol for V. natriegens, unfortunately this was not done as part of the work for this paper. From our experience, the efficiency is much lower than reported in the original paper. Often 2-3 dozen colonies from a single MuGENT reaction as opposed to the reported hundreds or more. Additionally, we often have to wait multiple days for the Vnat RifR colonies (the ones from Prof Dalia) to be visible. For the OAD in-situ complementation, for example, we co-transformed the OAD gene tDNA (250 ng) and the AmpR cassette tDNA (100 ng) (both were with 3 kb flanks). We got two AmpR colonies from overnight growth, and a total of 21 from another overnight incubation of the plate. All were AmpR. Of these, 5 were CmS – and these were all correct by colony PCR for both the AmpR cassette insertion and the in-situ complementation of OAD. As an aside, the co-transformation of the AmpR cassette had to be done because we did try in-situ complementation of just the OAD gene back in place where we inserted the CmR cassette, but we were not able to reasonably distinguish the colonies for which this worked (since we had to plate without antibiotics now). Although we did not characterize how many untransformed cells grew after these MuGENT transformations, it is a good idea for the future in order to keep track of efficiency and report in subsequent manuscripts.

717 should read "inoculated and grown overnight".

This adaptation has now been made (lines 934-935: "The indicated *V. natriegens* strains were inoculated and grown overnight from glycerol stocks at 30 °C in modified minimal medium").

720 I would personally write "diluted" rather than "re-inoculated".

This adaptation has now been made (lines 937-938: "The following morning, cells were diluted 1:200 in the same media and dispensed in triplicate into the wells of a BioScreen C Analyzer 100-well honeycomb plate").

734-735 The conversion factor and wavelength used to convert OD to cell count should be given.

The conversion factor is based on our flask experiments, in which we measured OD600 and CFU's for the specific media and salinity is 1.75 x 10^8 CFU ml^{-1} OD_{600}^{-1}. We have now provided this information in the manuscript (lines 952-955: "Based on the density of the overnight cultures, using a conversion factor of 1.75 x 10^8 CFU ml^{-1} OD_{600}^{-1}, the appropriate volumes containing ~10^5 cells were transferred to flasks containing pre-warmed modified M9 minimal medium with 1% (w/v) glucose and either 0 mM, 300 mM, 540 mM, or 650 mM NaCl.").

Fig 1 is a beautiful illustration of the workflow and results it presents.

Fig 2 is very clear.

Fig 3a The caption here could also mention that the sugar uptake rates were assumed to be the same as for glucose to make these model predictions -- this would help explain some of the discrepancies if the reader has skipped the text (which explains the results very clearly). Note that 3d and 3e referenced in the text are missing.

We have now calculated and measured biomass yields instead of uptake rate-based growth, so the suggested modification won't be necessary.

Fig 3c Should be 75.3% not 75,3%. The caption or the headers for the table could use a bit more information on the method for readers who have skipped the text, and especially there should be some clarification of what is meant by the different row titles in terms of growth prediction. It could be useful to present this data as a Venn diagram rather than a table.

Good suggestion. We have adapted the decimal notation and improved the description.

Fig 4 The title and captions could use a little more description in my view.

Good suggestion. We have now enhanced the description.

Fig 5b could be made larger relative to 5a. Otherwise this looks clear and effective.

Good suggestion. We have now increased figure 5b's relative size.

Reviewer #4:

I have reviewed the revised manuscript, the previous round of reviewer comments, and the point-by-point response of the authors. In this work, the authors present a metabolic network reconstruction for V. natriegens, an organism of considerable metabolic engineering interest due to its fast growth rate and possibility of natural competence. Thus, a metabolic model for the organism is of substantial interest. The authors manually curate a draft reconstruction, assess the accuracy of the model in predicting gene essentiality and growth on various substrate, production potential, and present an analysis of salt adaptation and resource allocation in the organism.

As the primary interest in the organism is metabolic engineering, understanding the production potential and high growth rate of the organism are of primary interest. In that respect, the authors are hitting all the right marks. I have no doubt that the basic metabolic functions of the model are reasonable (evidenced by central metabolic fluxes and comparison of active fluxes to observed proteomics data). However, I find that key outcomes of the work, both in model completeness and knowledge derived from the model analysis, are less than satisfying. While this is a valuable first step in understanding V. natriegens, I'm not sure it will catch a lot of interest in its current form without answering more fundamental questions about the potential of the organism for metabolic engineering, specifically around the origin of its high growth rate and possibility for production outside of well-studied pathways.

Major comments
- The model has only 858 metabolic genes (compared to 1515 in the latest E. coli reconstruction) and predicts only 44% of growth on substrates, despite a similar overall genome size to E. coli, indicating that it is missing a substantial amount of metabolic content still. This is also relevant for the assessment of production potential - the authors look at amino acids and biomass precursors, which presumably have well-annotated pathways that don't change much in yield across organisms, but production potential of a wider array of substrates would be more interesting to assess the genetically-encoded metabolic capabilities of this organism. However if the current annotation is still limited, this analysis seems to be unlikely to be successful.

We would like to thanks the reviewer for the constructive criticism.

To address the point raised by the reviewer regarding the low accuracy of carbon substrate prediction, we have now manually curated the Biolog data removing those substrates which are complicated to include in the model – such as substrates containing multiple chemical species like tween 20 - as it was done before for *E. coli* (https://bmcsystbiol.biomedcentral.com/articles/10.1186/1752-0509-8-79). The model then predicts 75% of growth on substrates (being 80% in E. coli). When negative results are included, the binary accuracy on substrate predictions is now 83.8%. (Figure 3b, lines 291-294: "The model predicted growth for 48 of 64 (75%) carbon sources on which *V. natriegens* can grow and predicted non-viability for 97 of 109 (89%) carbon sources on *which V. natriegens* cannot grow, leading to an overall predictive accuracy of 83.8%.).

While it is true that the last published model of *E. coli* has a larger number of genes, it is important to highlight that *V. natriegens* is a non-conventional organism, with only a handful of publications regarding genomic and metabolic information. This is very different from the most studied organism, *E. coli*, an organism with dozens of models available and lots of biochemical characterisation and systems biology data.

Our model has a comparable number of genes as other models of other no-so-well-studied gram-negative bacteria (see table below). In addition, we have performed extensive manual curation to reduce the number of genes in our model as suggested by reviewer 2 in the previous revision round, when we were encouraged to reduce rather than expand the number of genes/reactions, advice we followed by performing a stricter curation of our model by manually checking at all the reactions suggested by SEED that did not have any equivalents in the *Vibrio cholerae* model iAM-Vc960. As suggested by reviewer 2, more genes do not make the model better. The genes that have been removed in this last step of manual curation are included in Supplementary material so users can decide to work with an expanded version of the model.

Organism	Model	Number of genes	Reference
Komagataeibacter xylinus	iMR640	640	Rezazadeh et al. (2020)
Streptococcus oralis	iCJ415	415	Jensen et al. (2020)
Limosilactobacillus reuteri	iTN656		Namrak et al. (2022)
Mycobacterium tuberculosis	iEK1011	1011	Kavvas et al. (2018)
Acitenobacter baumanni	iLP844	844	Presta et al. (2017)
	iCN718	718	Norsigian et al. (2018)
Salmonella typhimurium	MetaSal	824	Hartman et al. (2014)
Staphylococcus aureus	iSA863	863	Mazharul Islam et al. (2020)
Streptococcus pneumoniae	iDS372	372	Dias et al. (2019)

In our work, we have expanded beyond *E. coli* metabolism by adding some of the most fundamental features of *V. natriegens* metabolism, such as the halophilic adaptations in the respiratory chain and an array of production pathways such as the one for ectoine production.
V. natriegens research is emerging rapidly, and that is why this contribution is significant in this field (while it may not be as useful for the *E. coli* community). We not only provide models that other researchers can use, but we also provide with the most extensive proteomic data available for this organism to date.

- The model performance stats are somewhat incomplete in their presentation (e.g. around line 307) and should include a full confusion matrix for essentiality and a more detailed breakdown of substrate-specific growth predictions and data. It currently seems like the authors are presenting the upside of their model performance, when a more unbiased presentation of performance would be preferred.

Due to the particular nature of the data used in this experiment, we originally decided not to include a confusion matrix, as it would not inform about the accuracy of model prediction, in fact, it would most likely confuse the reader. This is just because in *V. natriegens*, contrary to *E. coli* and other more studied organisms, there are no experimental gene essentially data.

The "gene essentiality" data that is used to compare the in silico predictions to was created using a CRISRPi genome-wide screen performed by Henry Lee from Harvard University (Lee et al., 2019). Therefore, this screen is not based in true knock-outs but in putative gene repression. This screen was only partially reproducible in Lee's study. Indeed, in two runs, Lee found 735 and 648 putative essential genes, of which 587 (92.4%) overlapped. Furthermore, the "core essentiality" of these 587 was determined by a comparison to gene essentiality data in *V. cholerae*. In the conclusion of his study, Lee still refers to this set of genes as "putative essential".

Because of these considerations, we do not see the value of a full confusion matrix, which would suggest that Henry Lee's data is an accurate measure of real/experimental gene essentiality.

Yet, due to the request for a confusion matrix by the reviewer, we have now added a confusion matrix as supplementary data (Supplementary file 6, lines 382-385: "A confusion matrix of this comparison is provided as Supplementary File 6. However, it is worth mentioning that Lee's CRISPRi screens rely on reduced expression rather than true knock-outs and a lack of exact reproducibility of the CRISPRi high-throughput screen was reported by Lee *et al*.").

- The comparison of model-predicted growth rates to data in Figure 3a seems flawed if I understand the

methodology correctly. In Line 225 the authors: "When a carbon-equivalent uptake rate to glucose is used as a constraint" - What is the basis for this assumption? It is best practice to measure the uptake rate of each substrate to make a quantitative growth rate prediction with FBA, or just make a binary growth/no-growth call.

This is a good point, and we recognise that our previous description of these results was flawed. What we should have compared with this approach and approximation to the "carbon-equivalent" approximation is the yields. In addition to 173 growth/no-growth type substrate assays, we have now performed additional experiments to assess *V. natriegens* biomass yields on five carbon substrates and compared them to model-predicted yields (entire section lines 319-358)

- The resource balance analysis (RBA) seems to be a very promising tool to understand the high growth rate of Vibrio compared to E. coli and other platform organisms, but I find the section to be lacking any real conclusions. The model was somewhat arbitrarily parameterized to enable the observed growth rates, but there does not seem to be any in vitro evidence that the default catalytic rate should be 619200 h-1, for example, so the exercise seems to become somewhat arbitrary. If there was suggestion of an actual mechanism for increased growth rate, ideally with some kind of validation, this section would be much more interesting, and indeed answering a critical question for this organism as it is being considered for metabolic engineering applications.

The model has been parametrized using our extensive proteomic data. However, it is common practise in RBA models to use a default catalytic rate value for those enzymes not detected experimentally. This has been done in Bulović et al., 2019 and Jahn et al., 2021.

Therefore, we used the mentioned value of 619200 h-1, taken from literature (Salvy & Hatzimanikatis, 2020). This Nature Communications paper made a thermodynamic growth model of *E. coli*'s metabolism and found an average value of kcat = 172 s^{-1} for all the known enzymes in that study. This value corresponds to the 619200 h^{-1} that we have used for our RBA model. Using this value is widespread and is also suggested as an option for the default catalytic rate in the published software from a more recent study using RBApy (Jahn et al., 2021).

Furthermore, it is important to stress that this default value was only used for the enzymes which were not detected experimentally in our proteomics assay. Indeed, in our work, all enzymes detected in the proteomics - aka the most highly expressed ones, covering most of the metabolic enzymes in *V. natriegens'* central metabolism - have custom calculated values.

We have tried to better clarify all this in the manuscript (lines 626-631: "The model was calibrated by estimating apparent catalytic rates for all the enzymes detected in our proteomics dataset using RBApy scripts. For the remaining enzymes which had not been detected in our proteomics data, a default catalytic rate had to be set. We used a rate of 619200 h^{-1} for this, based on the average kcat of 172 s^{-1} found for all *E. coli* enzymes studied by Salvy & Hatzimanikatis (2020).")

To expect the RBA simulations to yield an actual mechanism for increased growth rate is ambitious. While we did not unravel the reasons behind fast growth (we believe is not a single factor responsible), thanks to the comment of the reviewer, we revised the data obtained, and specifically the section about ribosomal resource allocation, which provides novel information on V. natriegens growth rates and physiology. We found that, contrary to other organisms, V. natriegens is seemingly able to increase growth rates without increasing the ribosomal fraction in the proteome and that V. natriegens can grow faster than E. coli with the same number of ribosomes in the proteome.

In the new version of the manuscript, we have collected all the novel insights we have gathered around fast growth and discuss them in an entirely re-written conclusion (entire section lines 695-770). Some of the novel findings covered in the conclusion are:

- Comparison of the predicted growth rates between iLC858 and the E. coli model iML1515 yielded no obvious significant advantages of the V. natriegens network to explain its exceptional growth rates. However, this analysis lead us to find that the V. natriegens enzymes Alanine dehydrogenase and L-alanine:glycoxylate aminotransferase, two genes not found on the E. coli genome, provide V. natriegens with a metabolic advantage over E. coli for the production of the amino acid glycine. These genes were also found to have high levels of transcription in a ribosomal profiling dataset of a previous study.
- Potential impact of the differences in the respiratory chain with E. coli in growth. The model suggested distinct features in the respiratory system of V. natriegenes, which are slightly more efficient in salty environments. The sodium transporters in V. natriegens respiratory system are a critical difference with E. coli's lifestyle. A major point of focus in our manuscript was the central role of the Na+-translocating oxaloacetate decarboxylase (Na+-OAD). Our model, proteomics data and knock-out experiment all suggest a role for this membrane pump under aerobic growth, to our knowledge the first indication of the active function of this enzyme during aerobic growth in bacteria.

- Our RBA model as well as proteomics data suggested an allocation of 22.6% amino acid cellular dry weight allocated to translational machinery in V. natriegens. This is similar to what has been found in E. coli yet E. coli does not reach equally fast growth rates for this ribosome allocation.
- Moreover, we did not find a significant correlation between ribosomal resource allocation and different growth rates across proteomics samples corresponding to three different salinities, as opposed to the expected increase in ribosomal resource allocation with increasing growth rates.

Minor comments
- The list of model manual curations in Supplementary File 1 has no justification, and with lack of human readable reaction identifiers, it is currently incomprehensible

This is a good point and we understand that our overview of manual curations lacked justification and human readability. We have now provided human readable names for all the reactions and genes in this file, as well as justifications.

- The authors claim a MEMOTE overall score of 90% - is there any breakdown of performance stats that would make this value more interpretable to the reader? What does this mean?

We have now provided the MEMOTE breakdown of this score as supplementary file 2 (lines 148-149: "A breakdown of this score can be found in Supplementary file 2.")

- The authors repeatedly compare to iJO1366 but iML1515 is the latest version of the E. coli reconstruction, out for several years now

We have now updated all comparisons to be with iML1515 (as well as sometimes still also with iJO1366). This did not change any of the outcomes of our comparisons. (lines 158-160: "Compared to the highly curated *E. coli* model iML1515, our model iLC858 is still limited in size: only 858 *V. natriegens* genes were accounted for in our model, compared to the 1515 *E. coli* genes accounted for in iML1515. ", entire section lines 420-475).

3rd Revision - Editorial Decision **31st Jan 2023**

Thank you for sending us your revised manuscript. We have now received the comment from the referee. As you will see below, the referee is overall satisfied with the modifications and thinks the study is now suitable for publication.

Before we can formally accept your manuscript, we would ask you to address the following editorial-level issues:

Reviewer #4:

I thank the authors for seriously considering my concerns and addressing them substantially. I understand the limitations of building a genome-scale model for a poorly annotated organism. I appreciate the addition of a confusion matrix and I don't necessary share the pessimism about using a CRISPRi screen as validation data, as traditional knockouts have their own issues (e.g. lack of adaptation to the condition and difficulty making growth/no growth calls). I find the expanded RBA section stimulating. I have no additional concerns.

Thank you again for sending us your revised manuscript. We are now satisfied with the modifications made and I am pleased to inform you that your paper has been accepted for publication.

EMBO Press Author Checklist

Corresponding Author Name: Dr. Rodrigo Ledesma-Amaro
Journal Submitted to: Molecular Systems Biology
Manuscript Number: MSB-2021-10523RRR

USEFUL LINKS FOR COMPLETING THIS FORM
The EMBO Journal - Author Guidelines
EMBO Reports - Author Guidelines
Molecular Systems Biology - Author Guidelines
EMBO Molecular Medicine - Author Guidelines

Reporting Checklist for Life Science Articles (updated January
This checklist is adapted from Materials Design Analysis Reporting (MDAR) Checklist for Authors. MDAR establishes a minimum set of requirements in transparent reporting in the life sciences (see Statement of Task: 10.31222/osf.io/9sm4x). Please follow the journal's guidelines in preparing your
Please note that a copy of this checklist will be published alongside your article.

Abridged guidelines for figures
1. Data
The data shown in figures should satisfy the following conditions:
- the data were obtained and processed according to the field's best practice and are presented to reflect the results of the experiments in an accurate and unbiased manner.
- ideally, figure panels should include only measurements that are directly comparable to each other and obtained with the same assay.
- plots include clearly labeled error bars for independent experiments and sample sizes. Unless justified, error bars should not be shown for technical
- if n<5, the individual data points from each experiment should be plotted. Any statistical test employed should be justified.
- Source Data should be included to report the data underlying figures according to the guidelines set out in the authorship guidelines on Data

2. Captions
Each figure caption should contain the following information, for each panel where they are relevant:
- a specification of the experimental system investigated (eg cell line, species name).
- the assay(s) and method(s) used to carry out the reported observations and measurements.
- an explicit mention of the biological and chemical entity(ies) that are being measured.
- an explicit mention of the biological and chemical entity(ies) that are altered/varied/perturbed in a controlled manner.
- the exact sample size (n) for each experimental group/condition, given as a number, not a range;
- a description of the sample collection allowing the reader to understand whether the samples represent technical or biological replicates (including how many animals, litters, cultures, etc.).
- a statement of how many times the experiment shown was independently replicated in the laboratory.
- definitions of statistical methods and measures:
  - common tests, such as t-test (please specify whether paired vs. unpaired), simple χ^2 tests, Wilcoxon and Mann-Whitney tests, can be unambiguously identified by name only, but more complex techniques should be described in the methods section;
  - are tests one-sided or two-sided?
  - are there adjustments for multiple comparisons?
  - exact statistical test results, e.g., P values = x but not P values < x;
  - definition of 'center values' as median or average;
  - definition of error bars as s.d. or s.e.m.

Please complete ALL of the questions below.
Select "Not Applicable" only when the requested information is not relevant for your study.

Materials

Newly Created Materials	Information included in the manuscript?	In which section is the information available? (Reagents and Tools Table, Materials and Methods, Figures, Data Availability Section)
New materials and reagents need to be available; do any restrictions apply?	Not Applicable	

Antibodies	Information included in the manuscript?	In which section is the information available? (Reagents and Tools Table, Materials and Methods, Figures, Data Availability Section)
For **antibodies** provide the following information:<br>- Commercial antibodies: RRID (if possible) or supplier name, catalogue number and or/clone number<br>- Non-commercial: RRID or citation	Not Applicable	

DNA and RNA sequences	Information included in the manuscript?	In which section is the information available? (Reagents and Tools Table, Materials and Methods, Figures, Data Availability Section)
**Short novel DNA or RNA including primers, probes**: provide the sequences.	Yes	Supplementary materials

Cell materials	Information included in the manuscript?	In which section is the information available? (Reagents and Tools Table, Materials and Methods, Figures, Data Availability Section)
**Cell lines:** Provide species information, strain. Provide accession number in repository **OR** supplier name, catalog number, clone number, and/**OR** RRID.	Not Applicable	
**Primary cultures:** Provide species, strain, sex of origin, genetic modification status.	Not Applicable	
Report if the cell lines were recently **authenticated** (e.g., by STR profiling) and tested for mycoplasma contamination.	Not Applicable	

Experimental animals	Information included in the manuscript?	In which section is the information available? (Reagents and Tools Table, Materials and Methods, Figures, Data Availability Section)
**Laboratory animals or Model organisms:** Provide species, strain, sex, age, genetic modification status. Provide accession number in repository **OR** supplier name, catalog number, clone number, **OR** RRID.	Not Applicable	
**Animal observed in or captured from the field:** Provide species, sex, and age where possible.	Not Applicable	
Please detail **housing and husbandry conditions**.	Not Applicable	

Plants and microbes	Information included in the manuscript?	In which section is the information available? (Reagents and Tools Table, Materials and Methods, Figures, Data Availability Section)
**Plants:** provide species and strain, ecotype and cultivar where relevant, unique accession number if available, and source (including location for collected wild specimens).	Not Applicable	
**Microbes:** provide species and strain, unique accession number if available, and source.	Yes	Materials and Methods

Human research participants	Information included in the manuscript?	In which section is the information available? (Reagents and Tools Table, Materials and Methods, Figures, Data Availability Section)
If collected and within the bounds of privacy constraints report on age, sex and gender or ethnicity for all study participants.	Not Applicable	

Core facilities	Information included in the manuscript?	In which section is the information available? (Reagents and Tools Table, Materials and Methods, Figures, Data Availability Section)
If your work benefited from core facilities, was their service mentioned in the acknowledgments section?	Not Applicable	

Design

Study protocol	Information included in the manuscript?	In which section is the information available? (Reagents and Tools Table, Materials and Methods, Figures, Data Availability Section)
If study protocol has been **pre-registered, provide DOI in the manuscript**. For clinical trials, provide the trial registration number **OR** cite DOI.	Not Applicable	
Report the **clinical trial registration number** (at ClinicalTrials.gov or equivalent), where applicable.	Not Applicable	

Laboratory protocol	Information included in the manuscript?	In which section is the information available? (Reagents and Tools Table, Materials and Methods, Figures, Data Availability Section)
Provide DOI OR other citation details if **external detailed step-by-step protocols** are available.	Yes	Materials and Methods

Experimental study design and statistics	Information included in the manuscript?	In which section is the information available? (Reagents and Tools Table, Materials and Methods, Figures, Data Availability Section)
Include a statement about **sample size** estimate even if no statistical methods were used.	Not Applicable	
Were any steps taken to minimize the effects of subjective bias when allocating animals/samples to treatment (e.g. **randomization procedure**)? If yes, have they been described?	Not Applicable	
Include a statement about **blinding** even if no blinding was done.	Not Applicable	
Describe **inclusion/exclusion criteria** if samples or animals were excluded from the analysis. Were the criteria pre-established?<br><br>If sample or data points were omitted from analysis, report if this was due to attrition or intentional exclusion and provide justification.	Not Applicable	
For every figure, are **statistical tests** justified as appropriate? Do the data meet the assumptions of the tests (e.g., normal distribution)? Describe any methods used to assess it. Is there an estimate of variation within each group of data? Is the variance similar between the groups that are being statistically compared?	Not Applicable	

Sample definition and in-laboratory replication	Information included in the manuscript?	In which section is the information available? (Reagents and Tools Table, Materials and Methods, Figures, Data Availability Section)
In the figure legends: state number of times the experiment was **replicated** in laboratory.	Yes	Figures
In the figure legends: define whether data describe **technical or biological replicates**.	Yes	Figures

Ethics

Ethics	Information included in the manuscript?	In which section is the information available? (Reagents and Tools Table, Materials and Methods, Figures, Data Availability Section)
Studies involving **human participants**: State details of **authority granting ethics approval** (IRB or equivalent committee(s), provide reference number for approval.	Not Applicable	
Studies involving **human participants**: Include a statement confirming that **informed consent** was obtained from all subjects and that the experiments conformed to the principles set out in the WMA Declaration of Helsinki and the Department of Health and Human Services Belmont Report.	Not Applicable	
Studies involving **human participants:** For publication of **patient photos**, include a statement confirming that consent to publish was obtained.	Not Applicable	
Studies involving experimental **animals**: State details of **authority granting ethics approva**l (IRB or equivalent committee(s), provide reference number for approval. Include a statement of compliance with ethical regulations.	Not Applicable	
Studies involving **specimen and field samples:** State if relevant **permits** obtained, provide details of authority approving study; if none were required, explain why.	Not Applicable	

Dual Use Research of Concern (DURC)	Information included in the manuscript?	In which section is the information available? (Reagents and Tools Table, Materials and Methods, Figures, Data Availability Section)
Could your study fall under dual use research restrictions? Please check biosecurity documents and list of **select agents and toxins** (CDC): https://www.selectagents.gov/sat/list.htm	Not Applicable	
If you used a select agent, is the security level of the lab appropriate and reported in the manuscript?	Not Applicable	
If a study is subject to dual use research of concern regulations, is the name of the **authority granting approval and reference number** for the regulatory approval provided in the manuscript?	Not Applicable	

Reporting

The MDAR framework recommends adoption of discipline-specific guidelines, established and endorsed through community initiatives. Journals have their own policy about requiring specific guidelines and recommendations to complement MDAR.

Adherence to community standards	Information included in the manuscript?	In which section is the information available? (Reagents and Tools Table, Materials and Methods, Figures, Data Availability Section)
State if relevant guidelines or checklists (e.g., **ICMJE, MIBBI, ARRIVE, PRISMA**) have been followed or provided.	Not Applicable	
For **tumor marker prognostic studies,** we recommend that you follow the **REMARK** reporting guidelines (see link list at top right). See author guidelines, under 'Reporting Guidelines'. Please confirm you have followed these guidelines.	Not Applicable	
For **phase II and III randomized controlled trials**, please refer to the **CONSORT** flow diagram (see link list at top right) and submit the CONSORT checklist (see link list at top right) with your submission. See author guidelines, under 'Reporting Guidelines'. Please confirm you have submitted this list.	Not Applicable	

Data Availability

Data availability	Information included in the manuscript?	In which section is the information available? (Reagents and Tools Table, Materials and Methods, Figures, Data Availability Section)
Have **primary datasets** been deposited according to the journal's guidelines (see 'Data Deposition' section) and the respective accession numbers provided in the Data Availability Section?	Yes	Data availability
Were **human clinical and genomic datasets** deposited in a public access-controlled repository in accordance to ethical obligations to the patients and to the applicable consent agreement?	Not Applicable	
Are **computational models** that are central and integral to a study available without restrictions in a machine-readable form? Were the relevant accession numbers or links provided?	Yes	Materials and Methods
If publicly available data were reused, provide the respective **data citations in the reference list**.	Not Applicable	
